# Mechanistic insights into neurotransmitter release and presynaptic plasticity from the crystal structure of Munc13-1 C_1_C_2_BMUN

**DOI:** 10.7554/eLife.22567

**Published:** 2017-02-08

**Authors:** Junjie Xu, Marcial Camacho, Yibin Xu, Victoria Esser, Xiaoxia Liu, Thorsten Trimbuch, Yun-Zu Pan, Cong Ma, Diana R Tomchick, Christian Rosenmund, Josep Rizo

**Affiliations:** 1 Department of Biophysics, University of Texas Southwestern Medical Center, Dallas, United States; 2 Department of Biochemistry, University of Texas Southwestern Medical Center, Dallas, United States; 3 Department of Pharmacology, University of Texas Southwestern Medical Center, Dallas, United States; 4 Department of Neurophysiology, NeuroCure Cluster of Excellence, Charité-Universitätsmedizin Berlin, Berlin, Germany; 5 Key Laboratory of Molecular Biophysics of the Ministry of Education, Huazhong University of Science and Technology, Wuhan, China; 6 College of Life Science and Technology, Huazhong University of Science and Technology, Wuhan, China; Max Planck Institute for Biophysical Chemistry, Germany

**Keywords:** neurotransmitter release, Munc13, presynaptic plasticity, synaptic vesicle fusion, calcium binding, Mouse

## Abstract

Munc13–1 acts as a master regulator of neurotransmitter release, mediating docking-priming of synaptic vesicles and diverse presynaptic plasticity processes. It is unclear how the functions of the multiple domains of Munc13–1 are coordinated. The crystal structure of a Munc13–1 fragment including its C_1_, C_2_B and MUN domains (C_1_C_2_BMUN) reveals a 19.5 nm-long multi-helical structure with the C_1_ and C_2_B domains packed at one end. The similar orientations of the respective diacyglycerol- and Ca^2+^-binding sites of the C_1_ and C_2_B domains suggest that the two domains cooperate in plasma-membrane binding and that activation of Munc13–1 by Ca^2+^ and diacylglycerol during short-term presynaptic plasticity are closely interrelated. Electrophysiological experiments in mouse neurons support the functional importance of the domain interfaces observed in C_1_C_2_BMUN. The structure imposes key constraints for models of neurotransmitter release and suggests that Munc13–1 bridges the vesicle and plasma membranes from the periphery of the membrane-membrane interface.

**DOI:** http://dx.doi.org/10.7554/eLife.22567.001

## eLife digest

The human brain contains billions of cells called neurons that communicate with each other using molecules called neurotransmitters. An electrical signal in one neuron triggers the release of neurotransmitters from the cell, which then activate or inhibit electrical signals in neighboring neurons. Inside the cell, neurotransmitters are stored in small bubble-like structures called synaptic vesicles. The vesicles fuse with the membrane that surrounds the cell to release the neurotransmitters. This process must be tightly controlled to ensure that neurotransmitters are released rapidly and at the right time.

A protein called Munc13 is a key component of the machinery that regulates the fusion of synaptic vesicles. It helps the synaptic vesicle to dock onto the cell membrane and get ready for fusion. Munc13 is a large protein and contains several different regions, including three domains called C_1_, C_2_B and MUN. These three domains control the release of neurotransmitters, but how they do so is poorly understood.

Xu, Camacho et al. used a technique called X-ray crystallography to analyse the three-dimensional shape of the part of Munc13 that contains the three domains. The experiments reveal that the MUN domain forms a long rod-like shape with the C_1_ and C_2_B domains packed at one end. Several mutations that reduce the ability of the domains to interact with each other altered the release of neurotransmitters from mouse neurons to different extents.

These findings suggest that the overall architecture of the region containing the C_1_, C_2_B and MUN domains is important for the normal activity of Munc13. The structure revealed by Xu, Camacho et al. sets a framework for understanding how Munc13 controls neurotransmitter release, and thus mediates diverse forms of information processing in the brain.

**DOI:** http://dx.doi.org/10.7554/eLife.22567.002

## Introduction

The release of neurotransmitters by synaptic vesicle exocytosis is critical for neuronal communication. This exquisitely regulated process involves several steps, including tethering of synaptic vesicles to specialized sites of the presynaptic plasma membrane called active zones, a priming step(s) that leaves the vesicles ready to release, and Ca^2+^-triggered membrane fusion (Südhof, 2013). Each of these steps can be modulated in a variety of presynaptic plasticity processes that underlie multiple forms of information processing in the brain (Regehr, 2012). The protein machinery that controls release (Rizo and Xu, 2015; Jahn and Fasshauer, 2012; Südhof and Rothman, 2009) includes the neuronal soluble N-ethylmaleimide-sensitive factor attachment protein receptors (SNAREs) synaptobrevin, syntaxin-1 and SNAP-25, which play a key role in membrane fusion by forming a tight four-helix bundle (the SNARE complex) that brings the vesicle and plasma membranes into close proximity (Söllner et al., 1993; Hanson et al., 1997; Poirier et al., 1998; Sutton et al., 1998). This complex is disassembled by N-ethylmaleimide-sensitive factor (NSF) and soluble NSF attachment proteins (SNAPs; no relation to SNAP-25) (Söllner et al., 1993) to recycle the SNAREs for another round of fusion (Mayer et al., 1996; Banerjee et al., 1996). The Sec1/Munc18 protein Munc18–1 and the large (200 kDa) active zone proteins called Munc13s are also crucial for release. Munc18–1 binds to a self-inhibited ‘closed’ conformation of syntaxin-1 (Dulubova et al., 1999; Misura et al., 2000) and orchestrates SNARE complex assembly in an NSF-SNAP-resistant manner together with Munc13, which helps to open syntaxin-1 (Richmond et al., 2001; Ma et al., 2011, 2013).

**Figure 1.**
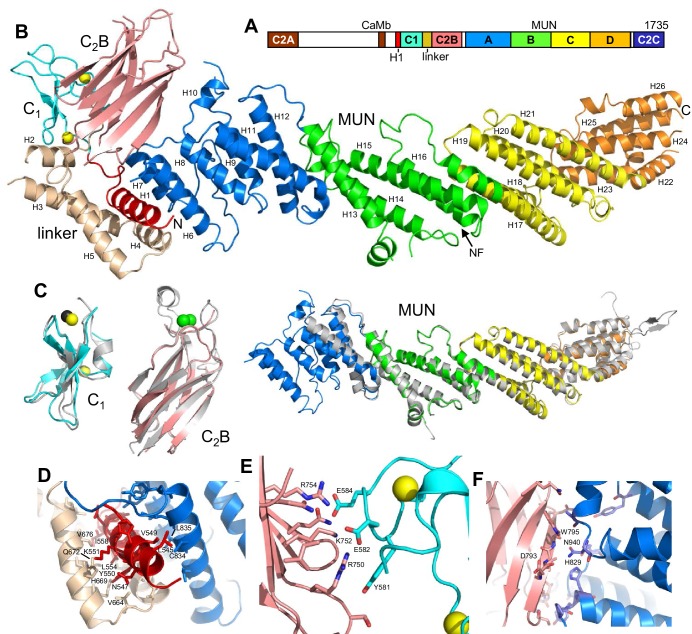
Crystal structure of Munc13–1 C_1_C_2_BMUN. (**A**) Domain diagram of rat Munc13–1, with A-D corresponding to the four subdomains of the MUN domain. CaMb = calmodulin-binding sequence. (**B**) Ribbon representation of the structure of Munc13–1 C_1_C_2_BMUN color-coded as in the domain diagram. Helices are numbered and labeled. The position of the NF sequence involved in opening syntaxin-1 is indicated. The Zn^2+^ ions bound to the C_1_ domain are shown as yellow spheres. (**C**) Superimposition of the structures of the Munc13–1 isolated C_1_ domain (PDB code 1Y8F), Ca^2+^-bound C_2_B domain (PDB code 3 KWU) and our refined structure of the almost complete MUN domain with the structures of these domains in the crystal structure of C_1_C_2_BMUN [r.m.s.d. between equivalent Cα atoms are 1.15 (45 Cα atoms), 0.35 (85 Cα atoms) and 0.96 (465 Cα atoms), respectively]. Ca^2+^ ions of the C_2_B domain structure are shown as green spheres. D-F. Interfaces of helix H1 with the linker helices and with the MUN domain (**D**), of the C_1_ domain with the C_2_B domain (**E**) and of the C_2_B domain with the MUN domain (**F**). Selected sides chains in the interfaces, including those that were mutated, are indicated. **DOI:** http://dx.doi.org/10.7554/eLife.22567.003

Because of their multiple functions, Munc13s have emerged as particularly central regulators of neurotransmitter release that link the core membrane fusion apparatus to diverse forms of presynaptic plasticity through their multidomain architecture (illustrated in Figure 1A for Munc13–1, the most abundant mammalian isoform). Thus, neurotransmitter release is completely abrogated in the absence of Munc13s (Augustin et al., 1999; Richmond et al., 1999; Aravamudan et al., 1999; Varoqueaux et al., 2002). This phenotype most likely arises because Munc13s play key roles in docking and priming (Varoqueaux et al., 2002; Weimer et al., 2006; Hammarlund et al., 2007; Imig et al., 2014) that are associated at least in part to their activity in opening syntaxin-1 and thus stimulating SNARE complex formation through their MUN domain (Richmond et al., 2001; Basu et al., 2005; Yang et al., 2015). However, the C_1_-C_2_B region and the C-terminal C_2_C domain are also critical for release, which may arise because these domains help bridge the vesicle and plasma membranes (Liu et al., 2016). There is also evidence for a role of Munc13s in events downstream of priming [e.g. (Hammarlund et al., 2007; Shin et al., 2010; Liu et al., 2016). Moreover, Munc13s are involved in multiple presynaptic plasticity processes, including isoform-specific depression and augmentation (Rosenmund et al., 2002), diacyglycerol (DAG)-phorbol ester-dependent potentiation of release via the C_1_ domain (Rhee et al., 2002) and Ca^2+^-dependent short-term plasticity through the C_2_B domain and a calmodulin-binding region (Shin et al., 2010; Junge et al., 2004). Binding of the Munc13–1 C_2_A domain to RIMs also provides a connection to RIM-dependent forms of short- and long-term presynaptic plasticity (Betz et al., 2001; Dulubova et al., 2005). The crucial physiological importance of these modulatory processes was emphatically illustrated by the finding that knock-in mice bearing a point mutation in the Munc13–1 C_1_ domain that disrupts phorbol-ester-dependent potentiation of release die 2–3 hr after birth even though the mutation causes no impairment of evoked release (Rhee et al., 2002).

**Figure 1—figure supplement 1.**
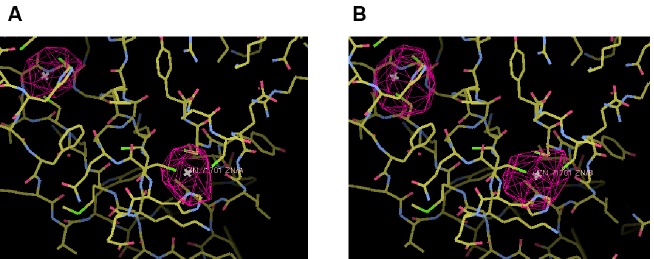
Zn^2+^ ions in the C_1_ domain of C_1_C_2_BMUN. Superimposed on the atoms is the anomalous difference map colored in magenta mesh and contoured at the 3σ level. The map was calculated from data with resolution limits of 49.9–6.0 Å, collected at the zinc K-absorption edge. (**A**) C_1_ domain of chain **A**. (**B**) C_1_ domain of chain B. **DOI:** http://dx.doi.org/10.7554/eLife.22567.004

**Figure 1—figure supplement 2.**
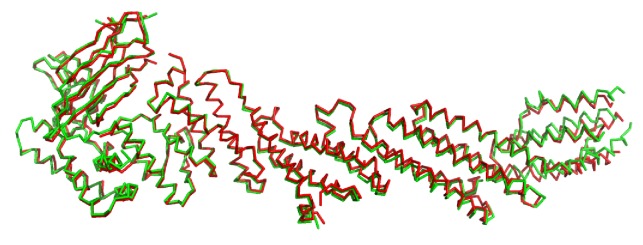
Comparison of chains A (green) and B (red) of C1C2BMUN. Superposition of 809 C-α carbon atoms resulted in an r.m.s.d. of 1.51 Å. **DOI:** http://dx.doi.org/10.7554/eLife.22567.005

**Figure 1—figure supplement 3.**
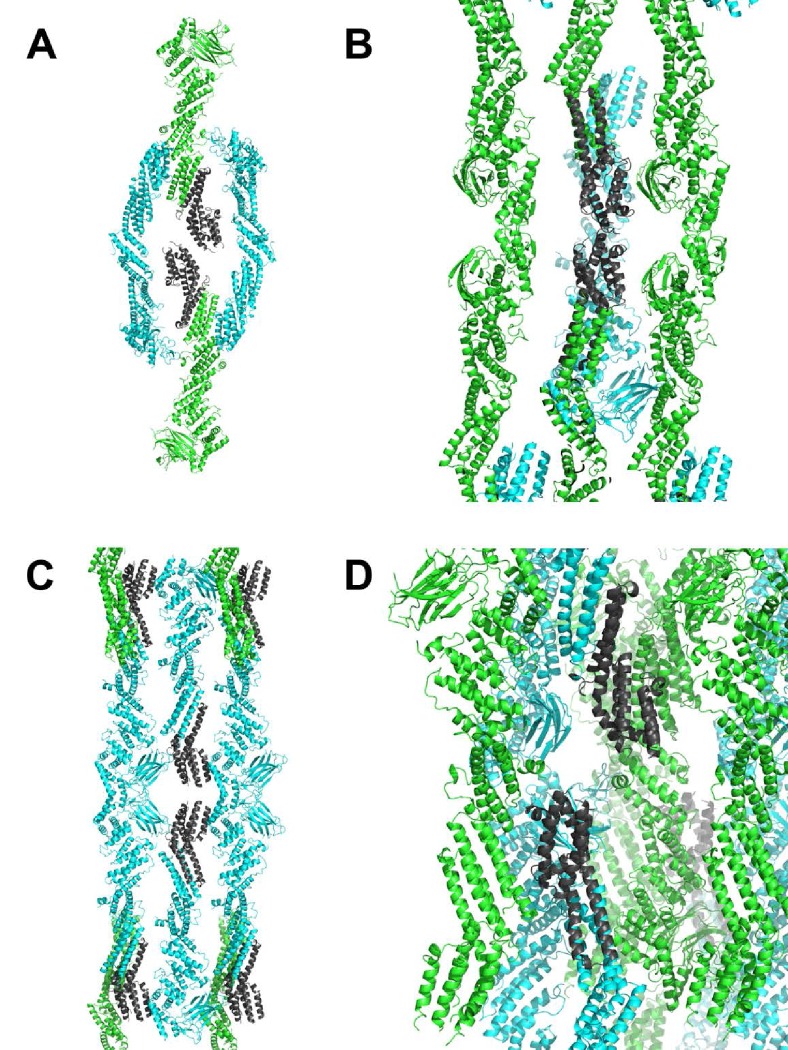
Lattice contacts for C1C2BMUN domains, C2 symmetry. Chain A is shown in green, chain B in cyan. In all panels, monomers in the foreground were removed for clarity. (**A, B**) Shown in dark grey are residues 1255–1517 of chain A. The view shown in **B** is rotated 90 degrees and zoomed in from that shown in **A**. (**C**, **D**) Shown in dark grey are residues 1255–1514 of chain **B**. The view shown in **D** is rotated 90 degrees and zoomed in from that shown in **C**. **DOI:** http://dx.doi.org/10.7554/eLife.22567.006

**Figure 1—figure supplement 4.**
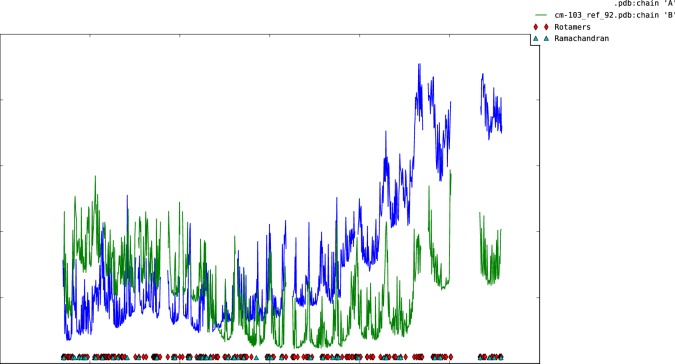
Plot of mean atomic displacement parameters (*B*-factors) versus residue number for the C1C2BMUN fragment. Chain A is shown in blue and chain B in green. **DOI:** http://dx.doi.org/10.7554/eLife.22567.007

**Figure 1—figure supplement 5.**
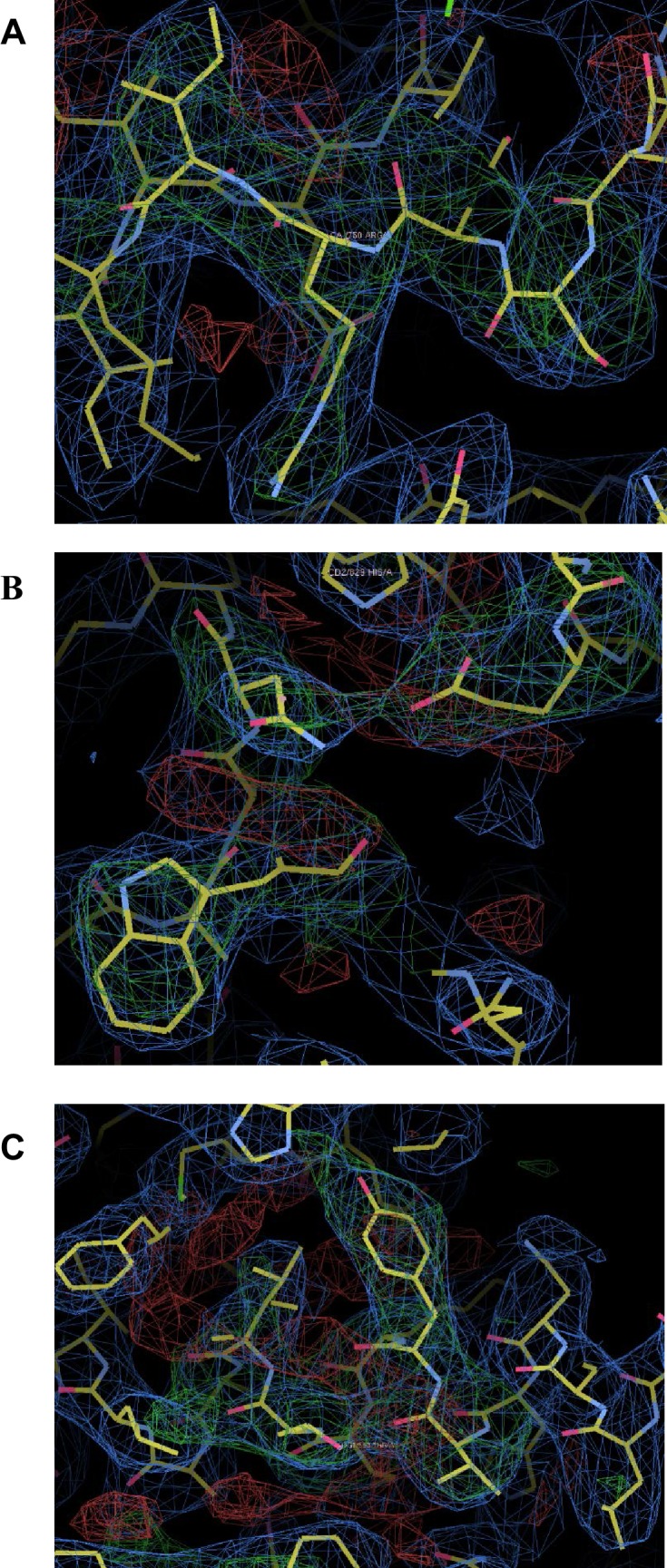
Omit maps for domain interfaces where mutations were made. (**A**) Superimposed on the refined coordinates for the C1C2BMUN structure is the omit map (mF_o_-DF_c_, green) contoured at the 3σ level. Residues A748-A752 (Ser748, Asp749, Arg750, Ile751, Lys752) were omitted from the C1C2BMUN structure in order to calculate the map. Shown in blue is the 2mF_o_-DF_c_ map (blue) contoured at the 1σ level. (**B**) Superimposed on the refined coordinates for the C1C2BMUN structure is the omit map (mF_o_-DF_c_, green) contoured at the 3σ level. Residues Trp795, Glu885 and Asn940 of chain A were omitted from the C1C2BMUN structure in order to calculate the map. Shown in blue is the 2mF_o_-DF_c_ map (blue) contoured at the 1σ level. (**C**) Superimposed on the refined coordinates for the C1C2BMUN structure is the omit map (mF_o_-DF_c_, green) contoured at the 3σ level. Residues Val549, Tyr550, Lys551, Lys552, Thr 553 and Leu554 of chain A were omitted from the C1C2BMUN structure in order to calculate the map. Shown in blue is the 2mF_o_-DF_c_ map (blue) contoured at the 1σ level. **DOI:** http://dx.doi.org/10.7554/eLife.22567.008

Despite these advances and the availability of three-dimensional structures for most of the Munc13 domains except C_2_C and part of the MUN domain (Shen et al., 2005; Dulubova et al., 2005; Rodríguez-Castañeda et al., 2010; Shin et al., 2010; Li et al., 2011; Yang et al., 2015), it is still unclear how the functions of these domains are related and coordinated, in part because no structure of a fragment containing multiple Munc13 domains has been described. Here, we report the crystal structure of a Munc13–1 fragment spanning its C_1_, C_2_B and MUN domains (C_1_C_2_BMUN), revealing a long, 195 Å rod formed by 26 α-helices that packs at one end against the C_1_ and C_2_B domains. The DAG-binding region of the C_1_ domain and the Ca^2+^-binding region of the C_2_B domain are near each other and point in the same direction, which is expected to facilitate cooperation between the two domains in membrane binding and thus enable synergy between the effects of DAG and Ca^2+^ in neurotransmitter release during repetitive stimulation. Electrophysiological experiments show that mutations designed to disrupt interfaces between different domains of C_1_C_2_BMUN impair evoked release and vesicle priming to different extents, and also have differential effects on Ca^2+^-dependent short-term plasticity as well as phorbol ester-induced potentiation. These results suggest that the highly elongated nature of C_1_C_2_BMUN and the relative disposition of its domains in our crystal structure are critical for the normal functions of Munc13–1 in neurotransmitter release and presynaptic plasticity, placing important structural constraints on possible models for the mechanisms of neurotransmitter release and presynaptic plasticity.

## Results

### Crystal structure of Munc13–1 C_1_C_2_BMUN

The work presented herein culminates 12 years of efforts dedicated to determine the three-dimensional structures of fragments encompassing part of or the entire highly conserved C-terminal region of Munc13–1, which includes the C_1_, C_2_B, MUN and C_2_C domains (Figure 1A). While crystals were obtained for several of the fragments that we prepared, they tended to diffract poorly. Key for the success in obtaining crystals of C_1_C_2_BMUN of sufficient quality to diffract in the 3–3.5 Å range were the choice of N- and C-termini (residues 529 and 1531, respectively), as well as the removal of residues 1408–1452, which correspond to a long loop within the MUN domain that is poorly conserved and is subject to alternative splicing (Brose et al., 1995). Removal of this loop generally increases the solubility of C-terminal Munc13–1 fragments (Ma et al., 2011; Li et al., 2011). Even with the best diffraction data obtained with C_1_C_2_BMUN, structure determination was hindered by low resolution, significant anisotropy, non-isomorphism and an inability to obtain selenomethionyl-derivatized protein. These problems were overcome by the use of single wavelength anomalous dispersion phases obtained from a dataset collected at the tantalum LIII edge on native crystals soaked with a tantalum bromide cluster, coupled with molecular replacement phases obtained from the previously determined structures of the C_1_ domain (Shen et al., 2005), the C_2_B domain (Shin et al., 2010) and the nearly complete MUN domain of Munc13–1. For this purpose, it was necessary to first re-refine the structure of the nearly complete MUN domain (Yang et al., 2015) using the deposited structure factors (PDB code 4Y21) to reduce the level of side chain outliers and of steric clashes, as well as to correct the sequence numbering (see Materials and methods). Placement of the C_1_ domains in the cell of the C_1_C_2_BMUN crystals (Figure 1—figure supplement 1) was verified by an anomalous difference map calculated from data collected on native crystals at the zinc K-edge energy. Data collection and refinement statistics for the final structure of C_1_C_2_BMUN, as well as for the re-refined structure of the nearly complete MUN domain, are described in Table 1.

**Table 1.** Data collection and refinement statistics. **DOI:** http://dx.doi.org/10.7554/eLife.22567.009

**Data collection**				
Crystal	Ta LIII-edge peak^*^	Zn K-edge peak^*^	MUN domain	Native
Space group	C2	C2	P2_1_2_1_2	C2
Cell constants (Å,°)	171.70 Å, 82.93 Å, 201.59 Å, 90.0°, 115.32°, 90.0°	174.72 Å, 84.55 Å, 202.10 Å, 90.0°, 115.11°, 90.0°	114.1 Å, 270.9 Å, 47.7 Å, 90.0°, 90.0°, 90.0°	176.13 Å, 86.34 Å, 202.13 Å, 90.0°, 115.54°, 90.0°
Wavelength (Å)	1.25489	1.28218	0.979	0.97931
Resolution range (Å)	42.73–4.50 (4.64–4.50)	49.81–4.00 (4.07–4.00)	39.02–2.90 (2.97–2.90)	45.60–3.35 (3.41–3.35)
Unique reflections	12,839 (620)	21,415 (961)	33,802 (2,770)	37,636 (1,363)
Multiplicity	3.6 (2.8)	3.8 (2.5)	5.4 (5.1)	3.9 (3.3)
Data completeness (%)	93.1 (64.4)	94.3 (86.4)	99.3 (98.7)	93.9 (68.9)
*R*_merge_ (%)^†^	3.1 (40.0)	10.1 (100.0)	6.9 (63.4)	5.6 (69.2)
*R*_pim_ (%)^‡^	1.8 (25.7)	5.7 (76.5)	NA	3.1 (38.8)
CC_1/2_ (outermost resolution shell)	0.974	0.490	NA	0.809
I/σ(I)	25.0 (0.8)	30.0 (2.1)	27.3 (1.95)	20.7 (1.3)
Wilson *B*-value (Å^2^)			85.4	104.7
Wilson *B*-value, sharpened (Å^2^)^§^	32.6	209.2	NA	49.6
**Refinement statistics**				
Resolution range (Å)			39.02–2.90 (2.98–2.90)	45.60–3.35 (3.46–3.35)
No. of reflections *R*_work_/R_free_			33,801/1,712 (2,634/136)	29,935/1,490 (752/34)
Data completeness (%)			99.2 (98.0)	75.3 (22.0)
Atoms (non-H protein/Zn^2+^/Cl^−^)			4286/NA/NA	13,597/4/2
*R*_work_ (%)			22.8 (34.5)	25.4 (32.7)
*R*_free_ (%)			25.3 (35.2)	29.0 (44.5)
R.m.s.d. bond length (Å)			0.002	0.003
R.m.s.d. bond angle (°)			0.499	0.610
Mean B-value (Å^2^) (chain A/chain B/ Zn^2+^/Cl^−^)			101.6/NA/NA	78.8/53.2/44.4/8.2
Ramachandran plot (%) (favored/additional/disallowed)^#^			95.3/3.9/0.8	92.1/6.7/1.2
Clashscore/Overall score^#^			2.57/1.37	3.51/1.62
Maximum likelihood coordinate error			0.38	0.41
Missing residues			933–941, 1041–1049, 1524–1531	A: 529–540, 704–707, 759–773, 801–807, 821–823, 923–928, 1038–1052, 1191–1196, 1342–1352, 1404–1469, 1518–1531. B: 529–541, 626-630, 703–708, 743–745, 759–774, 802–806, 820–824, 925–929, 1038–1050, 1338–1352, 1405–1467, 1516–1531.

Data for the outermost shell are given in parentheses.

^*^Bijvoet-pairs were kept separate for data processing.

^†^$R_{merge}=100\sum_{h}\sum_{i}|I_{h,i}-\left\langle I_{h}\right\rangle |/\sum_{h}\sum_{i}\left\langle I_{h,i}\right\rangle$, where the outer sum (h) is over the unique reflections and the inner sum (i) is over the set of independent observations of each unique reflection.

^‡^$R_{pim}=100\sum_{h}\sum_{i}[1/(n_{h}-1{)}^{1/2}]|I_{h,i}-\left\langle I_{h}\right\rangle |/\sum_{h}\sum_{i}\left\langle I_{h,i}\right\rangle$, where n_h_ is the number of observations of reflections **h**.

^§^*B*-factor sharpening was performed in the autocorrection mode of *HKL-3000* (Borek et al., 2013).

^#^As defined by the validation suite *MolProbity* (Chen et al., 2010).

The structures of the two molecules of C_1_C_2_BMUN present in the asymmetric unit of its crystals are very similar (Figure 1—figure supplement 2). The structure of C_1_C_2_BMUN can be viewed as a highly elongated rod spanning ca. 195 Å and is formed mostly of α-helical bundles, with a total of 26 helices; the C_1_ and C_2_B domains pack at the N-terminal end of the rod (Figure 1B). Much of the long rod is formed by the MUN domain, which is homologous to subunits of complexes that mediate tethering in diverse membrane compartments (Pei et al., 2009; Yu and Hughson, 2010) and was previously shown to be formed by four subdomains of about five helices each (named A-D and colored in blue, green, yellow and orange on Figure 1) (Li et al., 2011; Yang et al., 2015). Comparison of our C_1_C_2_BMUN structure with the crystal structure of the almost complete MUN domain (with the N-terminus at residue 933), which was described in Yang et al. (2015) and we re-refined, shows that the MUN domain is very similar in both structures (Figure 1C), as expected because this structure was used in crystallographic phase determination by the molecular replacement method. However, the structure of C_1_C_2_BMUN shows that the MUN domain actually starts at residue 828 and contains five additional helices (helices H6-H10), resulting in a total of seven helices for subdomain A (Figure 1C). In addition, there are five more helices preceding the MUN domain (helices H1-H5; Figure 1B). Four of these helices correspond to the linker sequence between the C_1_ and C_2_B domains, whereas one helix (H1) is spanned by a sequence preceding the C_1_ domain (colored in red).

These observations nicely explain a few findings made during our crystallization efforts. For instance, Munc13–1 fragments starting right at the beginning of the C_1_ domain were unstable and we were not able to express fragments containing only the C_1_ domain, the C_2_B domain and the linker between them in soluble form. As the structure of C_1_C_2_BMUN now reveals, there are extensive contacts between helix H1, the four helices of the linker, the MUN domain, the C_1_ domain and the C_2_B domain (Figure 1B,D–F), and hence it is not surprising that elimination of some of these interfaces impairs proper folding. We also note that the structures of the C_1_ and C_2_B domains in C_1_C_2_BMUN are similar to those determined for the isolated domains (Shen et al., 2005; Shin et al., 2010), although the Ca^2+^-binding loops are not visible in the C_2_B domain of C_1_C_2_BMUN (Figure 1C). This correlates with the previous finding that these loops were observable in the crystal structure of the C_2_B domain bound to Ca^2+^ but not in its crystal structure without Ca^2+^ (Shin et al., 2010), as our C_1_C_2_BMUN structure was determined in the absence of Ca^2+^.

**Figure 2.**
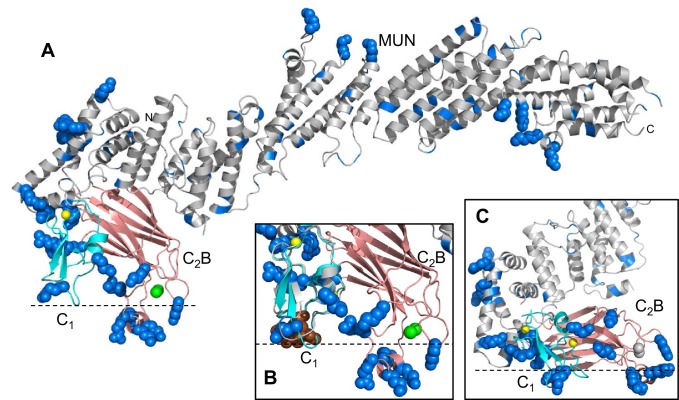
Distribution of basic residues clusters in C_1_C_2_BMUN. (**A**) Ribbon diagram of C_1_C_2_BMUN where the C_1_ and C_2_B domains were replaced with the structures of the isolated C_1_ domain (cyan; PDB code 1Y8F) and the isolated Ca^2+^-bound C_2_B domain (salmon; PDB code 3 KWU) (Shin et al., 2010) to include basic residues that are not observed in the structure of C_1_C_2_BMUN, likely because they are disordered. Arginine and lysine side chains are shown as blue spheres. The Zn^2+^ ions in the C_1_ domain are shown as yellow spheres and two Ca^2+^ ions bound to the C_2_B domain are shown as green spheres. Note that the two Ca^2+^ ions are not observed in the C_1_C_2_BMUN structure, which was crystallized in the absence of Ca^2+^. (**B**) Close up of the structure of C_1_C_2_BMUN as shown in A, but including a phorbol ester drawn as brown spheres. The position of the phorbol ester is based on superimposing the C_1_ domain of C_1_C_2_BMUN with the phorbol-ester bound structure of the PKC-δ C_1_B domain (PDB code 1PTR) (Zhang et al., 1995). The dashed line indicates the expected approximate location of the plasma membrane bound to the C_1_ domain through DAG and to the Ca^2+^-bound C_2_B domain through PIP_2_. (**C**) Close up of the same region of C_1_C_2_BMUN but shown in another orientation with a region containing multiple basic side chains (most from the C_1_ and C_2_B domains) at the bottom. The Ca^2+^-binding sites of the C_2_B domain are shown in gray to symbolize that the sites are not occupied. The dashed line indicates a potential localization of the plasma membrane resulting from binding of this basic face to PS, which is sufficient for binding of C_1_C_2_BMUN to liposomes even in the absence of Ca^2+^, DAG and PIP_2_ (Liu et al., 2016). **DOI:** http://dx.doi.org/10.7554/eLife.22567.010

The architecture of C_1_C_2_BMUN has important implications to understand the functions of Munc13–1 in docking and priming, as well as the regulatory roles of its various domains. First, the elongated structure and the overall packing of the different domains at the N-terminal end of the structure suggest that C_1_C_2_BMUN may function as a rigid or semi-rigid unit that bridges the synaptic vesicle and plasma membranes. Note in this context that the similarity in the structures of the two molecules from the asymmetric unit of the C_1_C_2_BMUN crystals (Figure 1—figure supplement 2) and between these structures and that of the nearly complete MUN domain (Figure 1C) suggest that the overall architecture of C_1_C_2_BMUN has limited flexibility. Second, a model of the structure of C_1_C_2_BMUN incorporating the Ca^2+^-binding loops of the C_2_B domain, which mediate its interactions with PIP_2_-containing membranes in a Ca^2+^-dependent manner (Shin et al., 2010), shows that these loops are proximal to the DAG-phorbol ester-binding region of the C_1_ domain (Figure 2A,B). This arrangement is expected to promote cooperation between the C_1_ and C_2_B domains in membrane binding, thus suggesting a natural mechanism for synergy between increases in DAG and intracellular Ca^2+^ concentrations to enhance release probability upon repetitive stimulation. However, it is noteworthy that there are abundant basic residues in the C_1_-C_2_B region (Figure 2A) that could potentially mediate membrane binding in alternative orientations in the absence of DAG and Ca^2+^-bound to the C_2_B domain (e.g. Figure 2C). These observations suggest that increased DAG and intracellular Ca^2+^ concentrations may alter release probability by promoting a different orientation of the Munc13–1 C-terminal region that is more efficient in promoting priming and/or fusion (see Discussion and models described therein). Third, the finding that helix H1 is packed against the MUN domain, with the N-terminus pointing toward the center of the long rod (Figure 1B), suggests that N-terminal sequences of Munc13–1 not included in this structure could perform their regulatory functions by influencing MUN domain activity. For instance, the calmodulin-binding region of Munc13–1 might inhibit release by binding to the middle of the MUN domain, where an NF sequence (Figure 1B) is known to be critical for opening syntaxin-1 (Yang et al., 2015), and binding of Ca^2+^-calmodulin to this region may enhance release by relieving this inhibition. Interactions of homodimerized Munc13–1 C_2_A domain (Lu et al., 2006) with the MUN domain might also underlie the inhibition caused by homodimerization, which is relieved by RIM binding (Deng et al., 2011).

### Functional consequences caused by disruption of C_1_C_2_BMUN domain interfaces

A key question that arises from the crystal structure of C_1_C_2_BMUN is whether the arrangement of the different domains at the N-terminal end of the structure is caused by crystal packing or reflects the native structure of Munc13–1 in neurons, and hence is important for its functions. To address this question, we designed three mutations to strongly disrupt domain interfaces observed in the C_1_C_2_BMUN structure: (i) a V549E,L554E double mutation designed to disrupt the hydrophobic packing of helix H1 against the linker region and against the MUN domain (Figure 1D); (ii) an R750E,K752E double mutation to disrupt salt bridges formed between the C_1_ and C_2_B domains (Figure 1E); and (iii) an N940W mutation designed to perturb the interface between the C_2_B and MUN domains (Figure 1F). We tested the physiological consequences of these mutations by performing rescue experiments in autaptic hippocampal neuronal cultures from Munc13-1/2 double KO mice (Varoqueaux et al., 2002) that express WT or mutant Munc13–1s through a lentiviral vector. To dissect which aspects of neurotransmitter release and its regulation might be affected by the mutations, we evaluated spontaneous release, Ca^2+^-dependent release triggered by a single action potential (AP), vesicle priming, high-frequency stimulation and PDBu-induced facilitation. Western blot analyses showed that the WT and mutant proteins were detectable at comparable levels (Figure 3—figure supplement 1). Although we cannot completely rule out the possibility that the differences observed in our electrophysiological measurements arise in part from distinct expression levels, this possibility is unlikely because no significant changes in synaptic properties are observed in heterozygous Munc13–1 (+/-) neurons and WT neurons mildly overexpressing WT munc13–1 in a Munc13–2 KO background (MC and CR, unpublished results).

**Figure 3.**
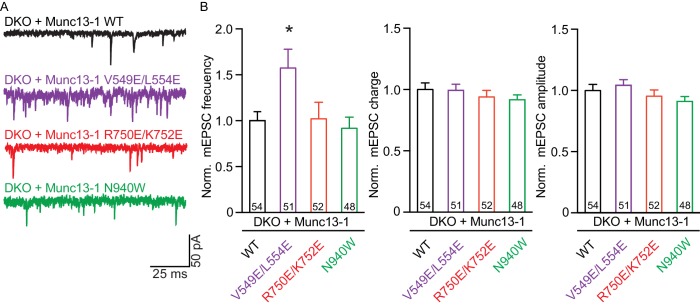
Effect on spontaneous release in synapses expressing Munc13–1 mutants that disrupt the C_1_C_2_BMUN domain interfaces. (**A**) Representatives traces of spontaneous release on synapses from Munc13-1/2 DKO rescued with the respective Munc13–1 WT and mutants indicated above. (**B**) Plots of mEPSC frequency, charge and amplitudes of Munc13–1 mutants normalized to corresponding Munc13–1 WT. Numbers in plots are *n* values for each group. Error bars represent SEM. Significance and p values were determined by comparison with the corresponding WT using the unpaired Student's *t* test: Mann-Whitney. *p<0.05. **DOI:** http://dx.doi.org/10.7554/eLife.22567.011

**Figure 4.**
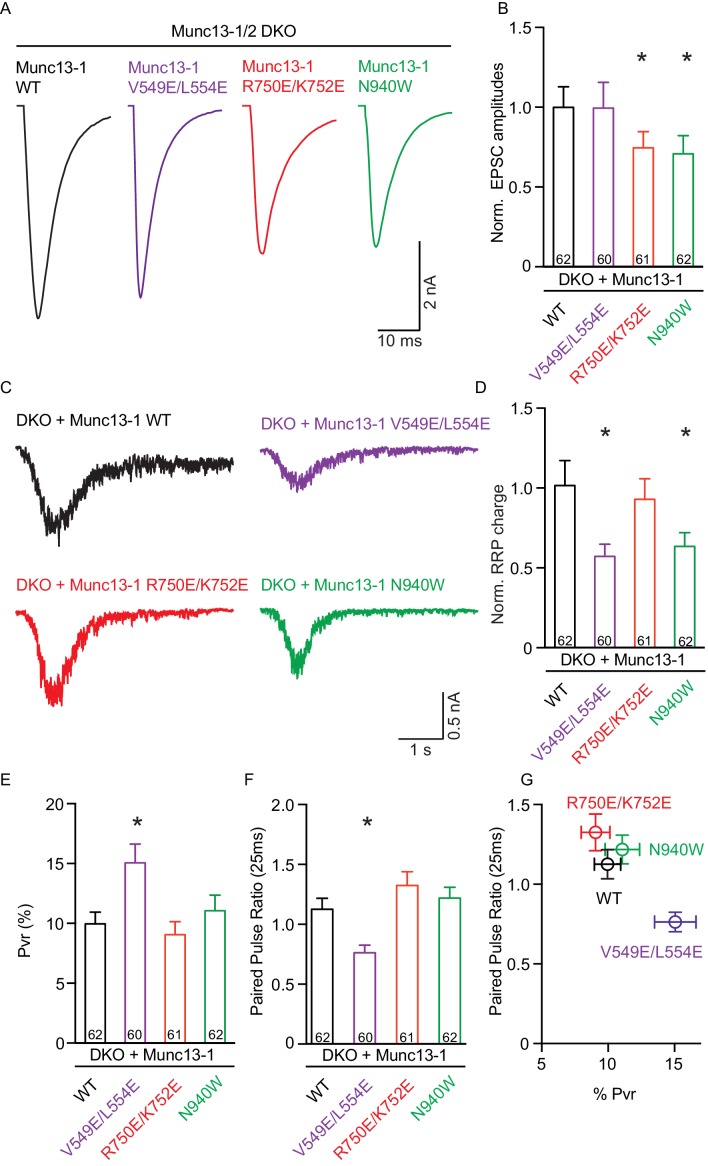
Single action potential evoked synaptic transmission, ready releasable pool and release probability consequences by the disruption of the C_1_C_2_BMUN domain interfaces. (**A**) Representatives traces of AP-evoked EPSC amplitudes recorded form Munc13-1/2 DKO and DKO neurons rescued with Munc13–1 WT and mutants as indicated above. (**B**) Plot of AP-evoked EPSC amplitudes of Munc13–1 disrupting C_1_C_2_BMUN domain interfaces mutants normalized to corresponding Munc13–1 WT. (**C**) Representative traces of synaptic responses induced by 500 mM sucrose from Munc13-1/2 DKO neurons rescued with the respective Munc13–1 WT and mutants indicated above. (**D**) Plot of RRP charge of Munc13–1 mutants normalized to corresponding Munc13–1 WT data. (**E**) Plot of *p*_vr_ for WT mutant Munc13–1s. (**F**) Plot showing the average paired-pulse ratios calculated from 2 AP-evoked EPSC amplitudes with a interstimulus interval of 25 ms. (**G**) Correlation between *p*_vr_ and paired-pulse ratios from DKO neurons rescued with Munc13–1 WT and mutants. Numbers in plots are *n* values for each group. Error bars represent SEM. Significance and p values were determined by comparison with the corresponding WT using the unpaired Student's *t* test: Mann-Whitney. *p<0.05. **DOI:** http://dx.doi.org/10.7554/eLife.22567.013

We first assessed the effects on spontaneous release measuring the frequency and the amplitude of miniature excitatory postsynaptic currents (mEPSCs). Only the V549E,L554E mutation in the N-terminal H1 helix showed an increase in mEPSC frequency, whereas the mEPSC charge and amplitude were unchanged (Figure 3). We next recorded excitatory postsynaptic currents (EPSCs) induced by a single AP to characterize evoked release. Analysis of the EPSC amplitudes revealed that the R750E,K752E mutation in the C_1_-C_2_B interface and the N940W mutation in the C2B-MUN interface impaired Ca^2+^-triggered release (Figure 4A,B). However, expression of the V549E,L554E mutant where helix H1 is perturbed fully rescued evoked release.

**Figure 3—figure supplement 1.**
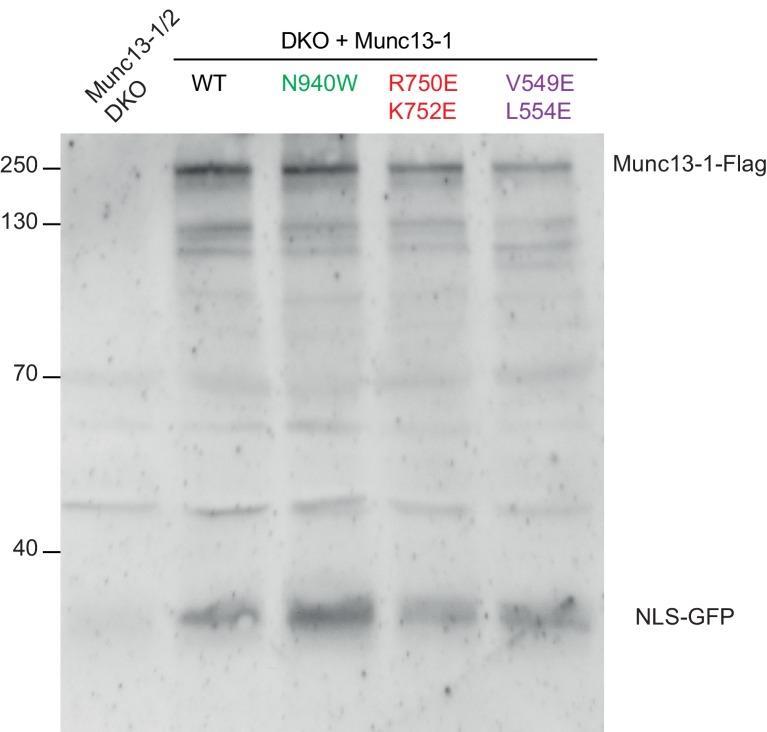
Detection of protein expression from Munc13-1/2 DKO hippocampal neurons. Sample immunoblot of Munc13-1/2 DKO neurons rescued with Munc13–1 WT or Munc13–1 point mutants indicated in the top. Signal at 250 kDa corresponds to the expected Munc13–1-flag, and the signal at around 30 kDa indicates the cleaved product NLS-GFP. Lane 1 shows the lack of expression of the protein Munc13–1-flag or NLS-GFP in untransfected Munc13-1/2 DKO neurons as a negative control. Lanes 2–5 show the protein expression of all Munc13–1 proteins used. 30 µg of proteins from the lysates were used. Molecular weights (kDa) are indicated on the left side. **DOI:** http://dx.doi.org/10.7554/eLife.22567.012

To dissect putative changes in the parameters that underlie evoked release, we first quantified the size of the readily releasable pool (RRP) by measuring the responses induced by hypertonic solution (Rosenmund and Stevens, 1996). We found that the N940W mutation in the C_2_B-MUN interface caused a decrease in RRP size of close to 40%, similar to the change seen in the evoked response. The H1 helix mutation V549E,L554E decreased the RRP size by nearly 50% (Figure 4C,D), which contrasts with the lack of an effect of this mutation on evoked release. Furthermore, the disruption of the C_1_-C_2_B interface by the R750E,K752E mutation led to normal vesicle priming despite reducing the evoked response. These experiments demonstrate that all three mutations impact Munc13–1 function but, interestingly, each mutation displays distinct effects on evoked release and vesicle priming. This finding strongly suggests that the domain interfaces differentially regulate the efficiency of the vesicle fusion process, which may lead to differences in vesicle release probability.

We calculated the vesicular release probability (Pvr) by dividing the EPSC charge by the charge of the RRP. The V549E,L554E mutation in the H1 helix increased the vesicular release probability significantly, as expected, while the small differences in Pvr observed for the R750E,K752E and N940W mutants with respect to WT were not statistically significant (Figure 4E). The impact on release probability in these mutants was further corroborated by analyzing the degree of facilitation or depression quantified by the paired pulse ratio of two consecutive AP-induced EPSC amplitudes (Figure 4F,G). Compared to WT, the V549E,L554E mutant showed more depression, in correlation with our finding of enhanced release probability, while the other two mutants showed similar paired-pulse behavior to WT rescues, in agreement with the conclusion that these mutations did not disrupt basal release probability substantially.

**Figure 5.**
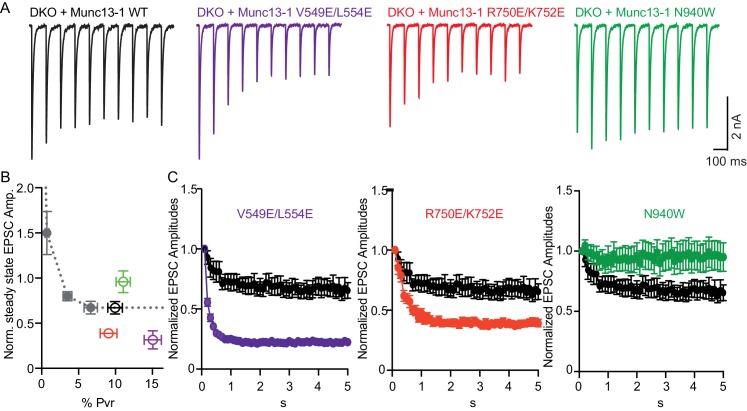
Difference in sustained release during a high-frequency action potential train upon disruption of C_1_C_2_BMUN domain interfaces. (**A**) Exemplary traces of EPSCs evoked by 10 Hz stimulation trains of Munc13-1/2 DKO neurons rescued with WT and mutant Munc13–1s. (**B**) Correlation between the amount of steady-state EPSC amplitudes at the end of the 10 Hz train and vesicular release probability. Dotted gray curve provides the correlation of steady-state depression and the release probability by applying AP trains at various external Ca^2+^ concentrations in WT neurons. (**C**) Analysis of 50 EPSC amplitudes evoked at 10 Hz, which were normalized to the first EPSCs and plotted over time. WT control (black circles) is identical in all three groups. **DOI:** http://dx.doi.org/10.7554/eLife.22567.014

In general, vesicular release probability is also a major determinant for release during sustained AP trains. Initial high vesicular release probability predicts more depression of synaptic responses due to more prominent depletion of primed vesicles. However, structure function studies on Munc13 have identified changes in sustained release that are not explained by the initial release probability, and this has been interpreted as Munc13 playing an additional role in synaptic augmentation (Rosenmund et al., 2002) and in modifying vesicle replenishment through activity-dependent vesicle re-priming (Shin et al., 2010). To test how disruption of the interfaces in Munc13–1 can affect the activity-dependent vesicle re-priming, we monitored synaptic responses during 10 Hz AP trains (Figure 5). We first defined the function of steady-state depression versus release probability in WT neurons by applying AP trains at various external Ca^2+^ concentrations, and found as expected a robust correlation between release probability and steady state depression (Figure 5B). Interestingly, the V549E,L554E mutation in the H1 helix and the R750E,K752E mutation in the C_1_-C_2_B interface caused more pronounced depression of sustained release than expected from the initial release probability, while disrupting the C_2_B-MUN interface with the N940W mutation caused less depression than expected from the initial release probability (Figure 5B,C). These results suggest that each one of the interfaces disrupted by the mutations plays a role in activity-dependent vesicle re-priming, and reinforce the notion that Munc13 exerts multiple functions in shaping release properties at synapses.

**Figure 6.**
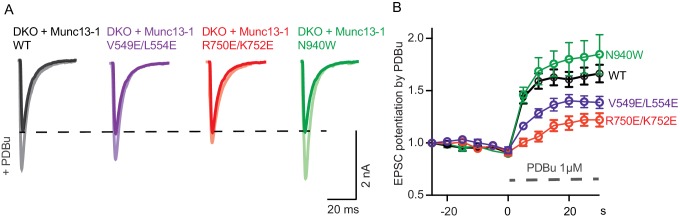
Effect of mutations that disrupt the C1C2BMUN domain interfaces on the potentiation of release caused by the activation of the C1 domain by phorbol ester. (**A**) Exemplary EPSC traces (dark colors) from WT and mutant groups and their corresponding EPSCs after PDBu application (light colors). (**B**) Potentiation of EPSC amplitudes by 1 μM PDBu evoked at 0.2 Hz in Munc13-1/2 DKO neurons expressing WT or mutants. The relative PDBu potentiation was calculated by normalizing the EPSC amplitude in PDBu with the presiding EPSC recorded in control extracellular solution. The solid symbols represent the normalized EPSC values in each time point (± SEM). **DOI:** http://dx.doi.org/10.7554/eLife.22567.015

The regulatory function of Munc13 can also be probed by applying phorbol esters that act as exogenous agonists of the C_1_ domain and increase the vesicle release probability. To test how the mutations in the domain interfaces of Munc13–1 alter the coupling of phorbol-ester binding to changes in release probability, we examined the effects of acute application of 1 µM PDBu, which causes a 60% increase in the evoked postsynaptic responses in WT neurons (Figure 6). The N940W mutation in the C_2_B-MUN interface showed more pronounced potentiation than WT Munc13–1 (Figure 6B), which correlates with the reduction in steady-state depression during 10 Hz AP trains observed for this mutant (Figure 5C) and indicates an easier transition for Munc13–1 to a potentiated state. Conversely, the V549E,L554E in helix H1 led to reduced potentiation by PDBu compared to WT, which likely arises because of the initial high vesicular release probability observed for this mutant (Figure 4E). Remarkably, the R750E,K752E mutation in the C_1_-C_2_B interface caused an even more pronounced decrease in PDBu potentiation (Figure 6B), which contrasts with the limited effect of this mutation on vesicular release probability (Figure 4E) but correlates with the strong depression observed in the 10 Hz AP trains (Figure 5C). These findings strongly support the notion that the interactions between the C_1_ and C_2_B domain observed in our crystal structure are critical for the interplay between the two domains in Ca^2+^- and DAG-dependent presynaptic plasticity.

**Figure 7.**
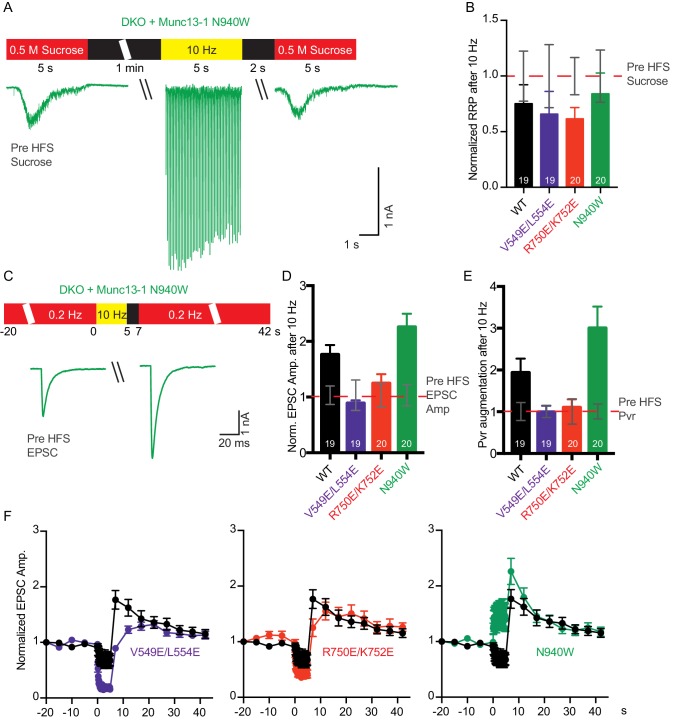
Effect of mutations that disrupt the C1C2BMUN domain interfaces on the potentiation of release caused by high-frequency stimulation. (**A**) Schematic diagram illustrating experimental design and example traces of synaptic responses induced by 500 mM sucrose from Munc13-1/2 DKO rescued with Munc13–1 N940W mutant before and after a 10 Hz action potential train. (**B**) Normalized summary plot of RRP charge of Munc13-1/2 DKO neurons rescued with the respective Munc13–1 WT and mutants indicated below. The RRP charges from the different groups were normalized to corresponding RRP charge recorded 1 min before the 10 Hz train (dashed red line). (**C**) Schematic diagram illustrating experimental design and example traces of EPSCs from Munc13-1/2 DKO rescued with Munc13–1 N940W mutant before and after a 10 Hz action potential train. (**D**) Normalized plot of AP-evoked EPSC amplitudes of Munc13-1/2 DKO neurons rescued with the respective Munc13–1 WT and mutants indicated below. The EPSC amplitudes were normalized to the corresponding EPSC recorded before the 10 Hz train (doted red line). (**E**) Plot of the estimated Pvr of Munc13-1/2 DKO neurons rescued with the respective Munc13–1 WT and mutants indicated below normalized to the corresponding Pvr before high-frequency stimulation (preHFS) (doted red line). (**F**) Normalized EPSC amplitudes of Munc13-1/2 DKO neurons rescued with the respective Munc13–1 WT and mutants indicated in each graph, in response to a low-frequency stimulus train (0.2 Hz) that is interrupted by a 5 s 10 Hz stimulus train, as outlined in panel (**C**). Numbers in plots are n values for each group. Error bars represent SEM. **DOI:** http://dx.doi.org/10.7554/eLife.22567.016

Because we observed changes in basal release probability and/or RRP size in the three mutants, we next examined how these release parameters are modulated during short-term plasticity induced by action potential trains. We recorded EPSC and sucrose-evoked responses before and 2 s after a 10 Hz action potential train to determine which of those parameters are individually affected (Figure 7). For rescues with WT Munc13–1 and the three mutants, the RRP size after the train tended to decrease compared to the RRP size before the train, but the difference was not significant (Figure 7B). On the other hand, the EPSC amplitude increased for the WT rescue after the train (Figure 7D,F), thus leading to an increased release probability with respect to the Pvr observed before the train (Figure 7E). These results indicate that in the WT rescues the train does not have a substantial effect on the RRP, but the efficiency of evoked release increases due to some alteration(s) of the release machinery.

In rescues with the V549E,L554E mutation in helix H1, comparison of RRP size and release probability before and after a 10 Hz train showed a failure to potentiate the RRP size, as observed in the WT rescue, but also a failure to increase the vesicular release probability (Figure 7B,E). These results contrast with the impairment of vesicle priming and increment of vesicular release probability observed for the V549E,L554E mutant in naive synapses (Figure 4D,E). Thus, the enhanced depression during the AP train observed for this mutant compared to WT Munc13–1 (Figure 5C) was due to a failure to potentiate release probability and not due to a failure in vesicle replenishment. Similar results were obtained for the rescue with Munc13–1 bearing the R750E,K752E mutation in the C_1_-C_2_B interface (Figure 7B,D–F), which in naïve synapses caused no impairment of RRP size and vesicular release probability (Figure 4D,E), but an enhanced depression of EPSC responses during AP trains (Figure 5C). Thus, this enhanced depression was not due to a failure in vesicle replenishment but to a failure to potentiate release probability, as observed for the V549E,L554E mutant. Finally, the N940W mutation in the C_2_B-MUN interface, which leads to a 40% reduction in RRP size (Figure 4D) but no change in release probability (Figure 4E) in naive synapses, caused a selective increase in vesicular release probability after the AP train, similar to what is seen in WT rescues but with larger degree of potentiation (Figure 7B,D–F). Thus, the finding that this mutant exhibits much less depression than WT during the AP train (Figure 5C) arises because this mutant is more likely to potentiate release probability during AP trains than WT Munc13–1.

Overall, our electrophysiological data provide compelling evidence for functional and structural interactions among the domains of the Munc13–1 C_1_C_2_BMUN fragment. These interactions play distinct roles in regulating vesicle priming, vesicle release probability and activity-dependent changes in release probability, thus having different impact on the ability of synapses to dynamically respond to incoming AP patterns.

### Effects of disruption of C_1_C_2_BMUN domain interfaces on reconstituted liposome fusion assays

Our reconstitution experiments, which monitor fusion between synaptobrevin liposomes (V-liposomes) and syntaxin-1-SNAP-25 liposomes (T-liposomes) in the presence of Munc18–1, NSF, αSNAP and a Munc13–1 fragment spanning its C_1_, C_2_B, MUN and C_2_C domains (C_1_C_2_BMUNC_2_C), recapitulate multiple features of neurotransmitter release, in particular the absolute requirement of Munc18–1 and Munc13–1 for membrane fusion (Liu et al., 2016). Hence, we used these experiments to examine the effects of disrupting Munc13–1 domain interfaces on membrane fusion in vitro and compare them to those observed in our electrophysiological studies. Unfortunately, multiple attempts to express C_1_C_2_BMUNC_2_C bearing the V549E,L554E mutation failed to yield sufficient amounts of soluble protein, and we were also unable to obtain C_1_C_2_BMUNC_2_C bearing a single V549E or L554E mutation, suggesting that both substitutions strongly destabilize the fragment. Since the V549E,L554E mutant was expressed at levels comparable to WT Munc13–1 in our rescue experiments, it is plausible that this mutation is less destabilizing in the context of these experiments because of interactions of that region with N-terminal sequences of Munc13–1, with other components of the release machinery, or with molecular chaperones.

**Figure 8.**
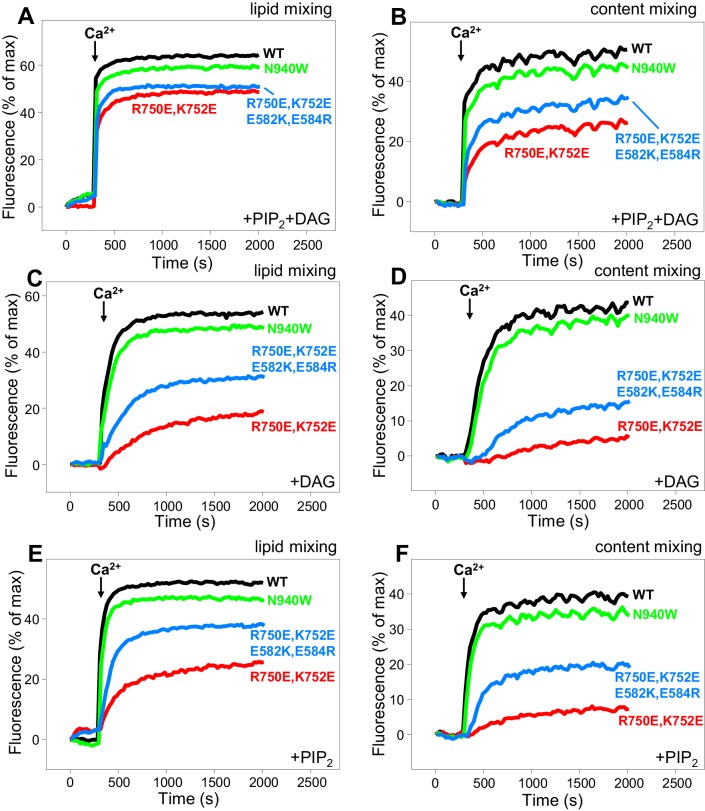
Effects of mutations that disrupt C_1_C_2_BMUN interfaces on membrane fusion in reconstitution assays. Lipid mixing (**A,C,E**) between V- and T-liposomes was measured from the fluorescence de-quenching of Marina Blue-labeled lipids and content mixing (**B,D,F**) was monitored from the development of FRET between PhycoE-Biotin trapped in the T-liposomes and Cy5-Streptavidin trapped in the V-liposomes. The assays were performed in the presence of Munc18–1, NSF-αSNAP and WT or mutant Munc13–1 C_1_C_2_BMUNC_2_C fragments as indicated. Experiments were started in the presence of 100 μM EGTA and 5 μM streptavidin, and Ca^2+^ (600 μM) was added after 300 s. **DOI:** http://dx.doi.org/10.7554/eLife.22567.017

In our reconstitution experiments, we use an assay that simultaneously measures lipid mixing and content mixing (Zucchi and Zick, 2011; Liu et al., 2016) to ensure that true membrane fusion is observed, as lipid mixing can occur without content mixing [e.g. (Chan et al., 2009; Zick and Wickner, 2014)]. We start the experiments in the absence of Ca^2+^, and Ca^2+^ is added after 300 s to examine its effect on liposome fusion. Under the standard conditions of our experiments, in which we use 500 nM C_1_C_2_BMUNC_2_C and we include DAG and PIP_2_ in the T-liposomes, fusion is highly efficient upon Ca^2+^ addition and the Ca^2+^ dependence arises from Ca^2+^ binding to the C_2_B domain (Liu et al., 2016). In initial experiments, we observed only mild effects of the R750E,K752E and N940W mutations in Munc13–1 C_1_C_2_BMUNC_2_C on fusion. To allow better discrimination, we lowered the C_1_C_2_BMUNC_2_C concentration to 100 nM, and we compared results obtained with T-liposomes that contained DAG+PIP_2_, DAG only, or PIP_2_ only (Figure 8). WT C_1_C_2_BMUNC_2_C was still highly active under all these conditions, supporting Ca^2+^-dependent membrane fusion that strictly required Munc13–1 C_1_C_2_BMUNC_2_C and was optimal when the T-liposomes included both DAG and PIP_2_ (Figure 8 and Figure 8—figure supplement 1). When the T-liposomes contained DAG and PIP_2_, the N940W mutation in the C_2_B-MUN interface slightly impaired fusion while the R750E,K752E mutation in the C_1_-C_2_B interface led to a considerably stronger impairment (Figure 8A,B; see Figure 8—figure supplement 2 for quantification). This impairment became more overt with T-liposomes containing only DAG or only PIP_2_, while the effects of the N940W mutation remained mild (Figure 8C–F).

**Figure 8—figure supplement 1.**
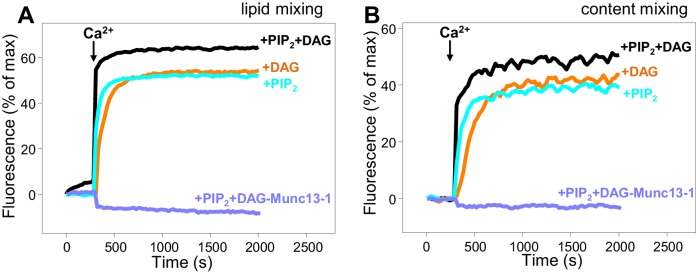
Dependence of liposome fusion on Munc13–1 C_1_C_2_BMUNC_2_C, DAG and PIP_2_. Lipid mixing (**A**) between V- and T-liposomes was measured from the fluorescence de-quenching of Marina Blue-labeled lipids and content mixing (**B**) was monitored from the development of FRET between PhycoE-Biotin trapped in the T-liposomes and Cy5-Streptavidin trapped in the V-liposomes. The T-liposomes contained DAG and PIP_2_, or DAG, or PIP_2_, as indicated. The assays were performed in the presence of Munc18–1, NSF-αSNAP and Munc13–1 C_1_C_2_BMUNC_2_C, except for a control lacking Munc13–1 C_1_C_2_BMUNC_2_C to show its requirement for membrane fusion. Experiments were started in the presence of 100 μM EGTA and 5 μM streptavidin, and Ca^2+^ (600 μM) was added after 300 s. **DOI:** http://dx.doi.org/10.7554/eLife.22567.018

**Figure 8—figure supplement 2.**
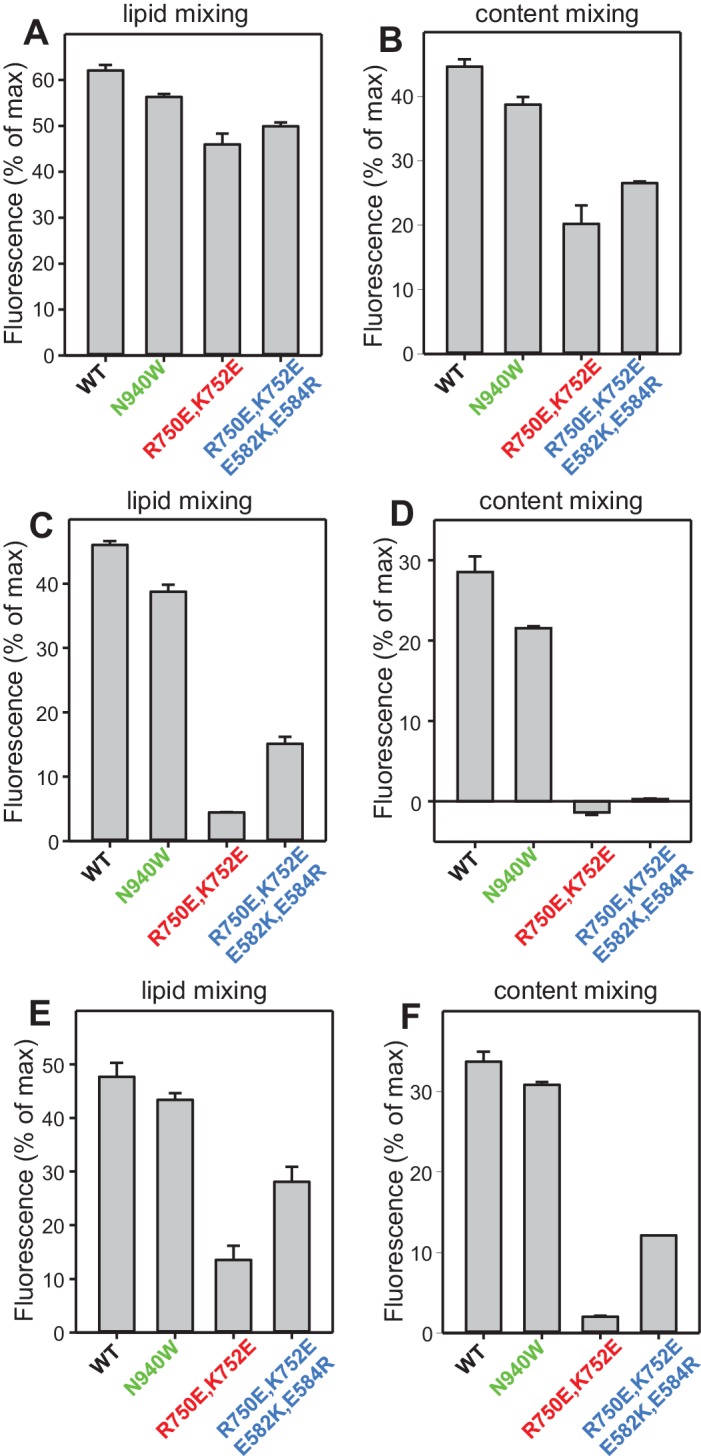
Quantification of the lipid and content mixing experiments of Figure 8. Panels (**A–F**) correspond to panels (**A–F**) of Figure 8, respectively. Bars represent averages of the normalized fluorescence observed after 500 s (200 s after Ca^2+^ addition) in experiments performed at least in triplicate. Error bars represent standard deviations. **DOI:** http://dx.doi.org/10.7554/eLife.22567.019

**Figure 8—figure supplement 3.**
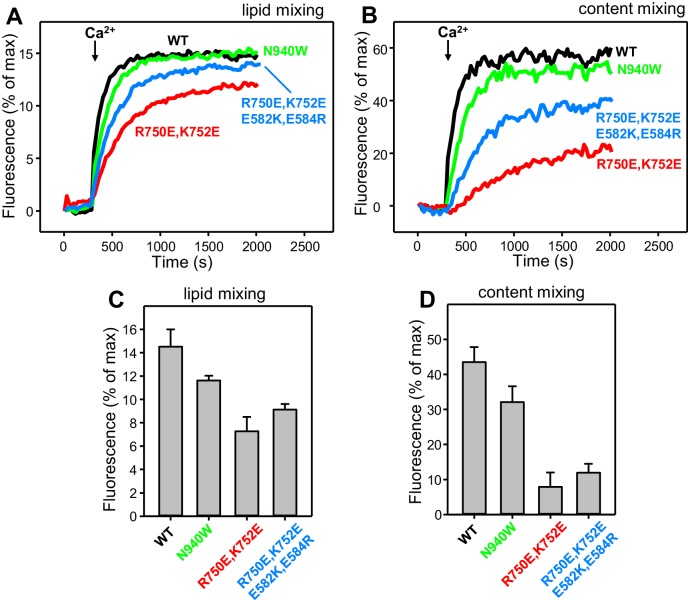
Effects of mutations that disrupt C_1_C_2_BMUN interfaces on membrane fusion in reconstitution assays including synaptotagmin-1. Lipid mixing (**A**) between V-liposomes containing synaptotagmin-1 and T-liposomes was measured from the fluorescence de-quenching of Marina Blue-labeled lipids and content mixing (**B**) was monitored from the development of FRET between PhycoE-Biotin trapped in the T-liposomes and Cy5-Streptavidin trapped in the V-liposomes. The assays were performed in the presence of Munc18–1, NSF-αSNAP and WT or mutant Munc13–1 C_1_C_2_BMUNC_2_C fragments as indicated. Experiments were started in the presence of 100 μM EGTA and 5 μM streptavidin, and Ca^2+^ (600 μM) was added after 300 s. (**C–D**) Quantification of the lipid and content mixing experiments of panels (**A–B**). Bars represent averages of the normalized fluorescence observed after 500 s (200 s after Ca^2+^ addition) in experiments performed at least in triplicate. Error bars represent standard deviations. **DOI:** http://dx.doi.org/10.7554/eLife.22567.020

To test whether the effects of the R750E,K752E mutation in these experiments are indeed due to disruption of the C_1_-C_2_B interface, we made a ‘reversal’ quadruple mutant where we mutated two of the acidic residues from the C_1_ domain in its interface with the C_2_B domain (Figure 1E) to basic residues (R750E,K752E,E582R,E584K). These two additional substitutions partially compensated for the effects of the R750E,K752E mutation, as the quadruple mutation recovered some of the activity lost in the R750E,K752E mutant (Figure 8). The recovery was modest, but this result is not unexpected because the orientation of the mutated side chains in the quadruple mutant may not be as favorable to form salt bridges as in the WT protein. Hence, the observed functional recovery in the quadruple mutant supports the notion that the effects of the R750E,K752E mutation in the fusion assays are due to disruption of C_1_-C_2_B interactions. We note that all these reconstitution experiments were performed in the absence of synaptotagmin-1 because this Ca^2+^ sensor does not have a marked influence in the results obtained in these bulk assays under the conditions used, although it may cause an acceleration of Ca^2+^-dependent fusion that can only be detected at faster time scales than those used in our measurements (Liu et al., 2016). Correspondingly, the relative activities of the WT and mutant C_1_C_2_BMUNC_2_C Munc13–1 fragments in reconstitution experiments that incorporated synaptotagmin-1 into the V-liposomes yielded similar results (Figure 8—figure supplement 3) to those obtained in the absence of synaptotagmin-1 (Figure 8A,B).

The impairment of fusion caused by the R750E,K752E in our reconstitution assays correlates with the impairment of evoked release observed in the rescue experiments, as well as with the strong depression observed for this mutation in the 10 Hz trains and the impairment of PDBu-induced augmentation (Figures 4–6). Altogether, our results strongly support the notion that the C_1_-C_2_B interface observed in the crystal structure of C_1_C_2_BMUN is important for evoked release and for cooperation between the C_1_ and C_2_B domains in membrane binding during repetitive stimulation to generate an activated state that is stabilized by binding of the C_1_ domain to DAG and of the C_2_B domain to Ca^2+^-PIP_2_. Thus, as we noted previously (Liu et al., 2016), our reconstitutions including DAG and PIP_2_ in the T-liposomes may recapitulate more closely this activated state than the normal state that leads to release evoked by a single action potential. Interpreting the results of the N940W mutation in the reconstitutions is hindered by the fact that this mutation impaired evoked and sucrose-induced release but had less depression than WT in the 10 Hz trains and a slightly higher potentiation by PDBu (Figures 4–6). The small impairment of fusion observed in the reconstitutions for this mutant may reflect a balance between the inhibitory and stimulatory effects observed in the rescue experiments but, overall, the mild nature of the effect on fusion appears to be more reminiscent of the small (albeit contrary) effects observed in the PDBu treatment, thus supporting also the notion that the reconstitutions emulate an activated state of the release machinery.

## Discussion

Munc13–1 acts as a master regulator of neurotransmitter release, playing a crucial role in release itself and multiple functions in integrating the effects of diverse signals that alter release probability during presynaptic plasticity. Thus, elucidating how the functions of the multiple domains of Munc13–1 are coordinated to control release and plasticity constitutes a major, fundamental challenge in neuroscience. The crystal structure of Munc13–1 C_1_C_2_BMUN and the functional data presented here represent critical steps to meet this challenge, revealing a highly elongated structure that is key to understanding how Munc13–1 bridges the vesicle and plasma membranes to control SNARE complex formation, and how the C_1_ and C_2_B domains mediate presynaptic plasticity processes that depend on Ca^2+^ and DAG.

Notable features of the structure of C_1_C_2_BMUN include its length, which is close to 20 nm and is thus comparable to the radius of a synaptic vesicle, and the interfaces between its different domains. The observation that the three mutations designed to disrupt domain interfaces observed in the structure have distinct effects on evoked release, vesicle priming, vesicle release probability, high-frequency stimulation and PDBu-induced facilitation demonstrates the functional relevance of the overall structure and shows that the different domain interfaces perturbed by the mutations play differential roles in release and short-term presynaptic plasticity. This conclusion is further supported by the observation that each of the three mutations uniquely alters the normal relation between vesicle release probability and steady-state EPSC amplitude in 10 Hz AP trains in neurons expressing WT Munc13–1 (Figure 5B), a relation that arises from the natural increase in RRP depletion rate as vesicle release probability increases. Similarly, the three mutations also change the normal inverse relation between the extent of PDBu-induced facilitation and the vesicle release probability. This finding is not surprising, as the effects of PDBu are likely to be at least partially related to those observed in 10 Hz trains. Thus, phorbol ester activation of the Munc13 C_1_ domain is believed to mimic the effects of increased DAG levels during repetitive stimulation, which result from accumulation of intracellular Ca^2+^ and activation of PLCs (Rhee et al., 2002). Accumulation of Ca^2+^ also activates Munc13s by binding to the C_2_B domain (Shin et al., 2010).

**Figure 9.**
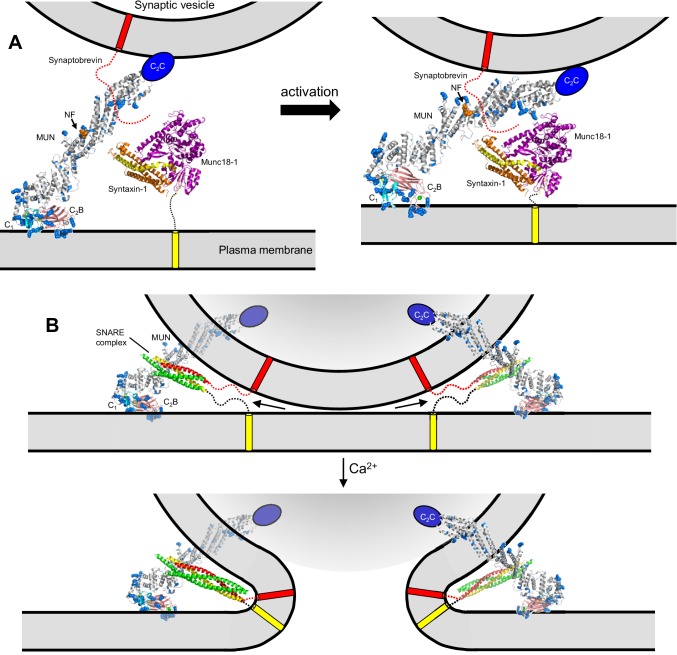
Models of neurotransmitter release inspired by the structure of C_1_C_2_BMUN and our functional data. (**A**) C_1_C_2_BMUNC_2_C is represented by the structure of C_1_C_2_BMUN, with the C_2_B domain replaced by the structure of the isolated Ca^2+^-bound C_2_B domain (colored in salmon), and by a blue ellipse corresponding to the C_2_C domain. C_1_C_2_BMUNC_2_C is shown bridging the two membranes through interactions of the vesicle membrane with the C_2_C domain and the plasma membrane with the basic surface formed by the C_1_ and C_2_B domains (left panel) or with the DAG-binding region of the C_1_ domain and the Ca^2+^-binding region of the C_2_B domain (right panel) (see Liu et al., 2016). Arginine and lysine side chains are shown as blue spheres. The C_1_ domain ribbon is in cyan and Zn^2+^ ions are shown as yellow spheres. The two Ca^2+^ ions bound to the C_2_B domain are shown as green spheres on the right; on the left, they are shown as gray spheres to represent that the sites are not occupied. The NF sequence involved in opening syntaxin-1 (Yang et al., 2015) is represented by orange spheres. The closed syntaxin-1-Munc18–1 complex (PDB code 3C98) is shown to scale to allow comparison of its size with that of C_1_C_2_BMUN. The C-terminus of the syntaxin-1 SNARE motif and the cytoplasmic region of synaptobrevin are shown as dashed curved lines, and their transmembrane regions are represented by cylinders. B. C_1_C_2_BMUNC_2_C is represented as in A but, instead of being located between the two membranes, is bridging the two membranes from a peripheral location. The model must be visualized in three dimensions, such that the C_2_C domain is bound to the outer surface of the vesicle membrane rather than inserted into the vesicle lumen. On the right side, the C_2_C domain is in the front, whereas in the left side the C_2_C domain is in the back of the vesicle, which is represented by a semitransparent gray surface. In both panels, C_1_C_2_BMUNC_2_C is shown in the orientation proposed for the activated state induced during repetitive stimulation, where binding to the plasma membrane is mediated by the DAG-binding region of the C_1_ domain and the Ca^2+^-binding region of the C_2_B domain. The SNARE complex (PDB code 1N7S) (Ernst and Brunger, 2003), with the SNARE motifs in green for SNAP-25, in red for synaptobrevin and in yellow for syntaxin-1, is shown partially assembled at the top and fully assembled at the bottom. The arrows in the top panel are meant to illustrate that complete assembly of the C-terminus of the SNARE complex might pull the membranes radially outwards, which could create strong membrane tension to trigger membrane fusion. See text for further details. **DOI:** http://dx.doi.org/10.7554/eLife.22567.021

The finding that the DAG/phorbol ester-binding region of the C_1_ domain and the Ca^2+^-binding loops of the C_2_B domain are close to each other and point in the same direction in our C_1_C_2_BMUN structure (Figure 2) suggest that Ca^2+^, DAG and PIP_2_ can cooperate in inducing binding of Munc13–1 to the plasma membrane, indicating that these different forms of activation are closely interrelated. These conclusions are supported by the physiological effects of the R750E,K752E mutation in the C_1_-C_2_B interface. This mutation leads to a modest impairment of evoked release and to no significant change in the RRP, resulting also in no significant change in vesicular release probability (Figure 4). However, this mutation causes a considerably stronger depression during 10 Hz trains than observed for WT Munc13–1 and to severe impairment of PDBu-dependent facilitation (Figures 5 and 6). These results suggest that the interaction between the C_1_ and C_2_B domains observed in our crystal structure is important for evoked release but is particularly critical for activation of Munc13–1 by phorbol esters as well by Ca^2+^ and DAG during repetitive stimulation. Note also that this mutation strongly disrupts the ability to increase the release probability after repetitive stimulation observed for WT Munc13–1 (Figure 7E). Because the C_1_ and C_2_B domains pack at the N-terminal end of the long helical structure, far from the middle region of the MUN domain that contains the NF residues involved in opening syntaxin-1 (Yang et al., 2015) (Figure 1B), our structure does not support models whereby the C_1_ and C2B domains impair the activity of the MUN domain by direct intramolecular interactions, and binding to DAG or Ca^2+^ releases these inhibitory interactions [e.g. (Rizo and Rosenmund, 2008)]. Instead, our structure suggests that activation of Munc13–1 by PDBu or by repetitive stimulation involves cooperative binding of the C_1_ and C_2_B domains to the plasma membrane in a defined orientation that is promoted by increases in the levels of DAG and intracellular Ca^2+^; this activated state may be more effective in mediating priming and/or downstream events leading to fusion than the state existing under resting conditions, which may involve a different orientation of Munc13–1 with respect to the plasma membrane favored by Ca^2+^- and DAG-independent interactions involving multiple basic residues of the C_1_ and C_2_B domains (Figure 2C; see also Figure 9 and discussion below).

The V549E,L554E mutation designed to disrupt the packing of helix H1 against the linker sequence and the MUN domain decreases the RRP but not the amplitude of the EPSCs induced by a single action potential, resulting in an increased vesicle release probability (Figure 4) that mirrors an enhancement in spontaneous release (Figure 3). Correspondingly, this mutant exhibits more depression during 10 Hz trains than WT neurons, but the depression is stronger than expected from the observed vesicle release probability (Figure 5B). The V549E,L554E mutation also leads to a strong impairment in PDBu-induced facilitation (Figure 6) that likely arises from the increased Pvr. Overall, the effects of this mutation resemble those caused by the H567K mutation that is expected to unfold the C_1_ domain (Rhee et al., 2002; Basu et al., 2007). The decreases in the RRP observed for both the V549E,L554E and the H567K mutants may result from disruption of coupling between the C-terminal region and the N-terminus containing the C_2_A domain, which contributes to the docking-priming activity of Munc13–1 (MC and CR, unpublished results). The increased release probability observed for both mutants suggests that the disruptive structural effects of the two mutations may mimic to some extent the changes involved in activating Munc13–1 during repetitive stimulation, which may involve in part the release of inhibitory effects caused by N-terminal sequences. However, note that the V549E,L554E mutation prevented the increase in Pvr observed after repetitive stimulation for WT Munc13–1 (Figure 7E), indicating that different features govern this increase in Pvr and the release probability in naïve synapses.

The N940W mutation in the C_2_B-MUN interface impairs evoked release and decreases the RRP, leading to a release probability similar to that of WT Munc13–1 (Figure 4). However, this mutant exhibits smaller depression during 10 Hz trains than WT Munc13–1 and a slightly larger PDBu-induced facilitation (Figures 5 and 6). These results suggest that the packing of the C_2_B-MUN interface observed in our structure is important for normal vesicle priming and release caused by a single action potential, but the alteration of this interface caused by the N940W mutation favors the activated state that is generated during repetitive stimulation. This view is reinforced by the finding that the N940W mutation causes a larger increase in release probability after repetitive stimulation than that observed with WT Munc13–1 (Figure 7E).

The neurotransmitter release machinery is highly complex, including several large proteins of hundreds of kDa that form the active zone in addition to the SNARES, Munc18–1, Munc13–1, NSF, αSNAP, synaptotagmin-1 and multiple additional components (Südhof, 2013). Without knowing how Munc13–1 interacts with other proteins, interpretation of the structure of Munc13–1 C_1_C_2_BMUN in terms of specific models of neurotransmitter release is necessarily speculative. Nevertheless, the structure poses critical constraints on working models of release and its regulation. For instance, the model of Figure 9A is based on the finding that the Munc13–1 C_1_C_2_BMUNC_2_C fragment bridges V-liposomes to T-liposomes in the absence of Ca^2+^ (Liu et al., 2016). Such bridging might facilitate the activity of the MUN domain in opening syntaxin-1 to initiate SNARE complex formation and may involve interactions of the polybasic face of the C_1_-C_2_B region (Figure 2C) with the T-liposomes and of C_2_C with the V-liposomes. Binding of the C_1_ domain to DAG and of the C_2_B domain to Ca^2+^ during repetitive stimulation could change the orientation of Munc13–1 with respect to the plasma membrane (Figure 2B) in such a way that the two membranes are brought into closer proximity (Figure 9A) and the SNARE complex is formed more efficiently. Importantly, if Munc13–1 is located between the two membranes, progress toward complete SNARE complex assembly and fusion would require release of Munc13–1 from the fusion complex because otherwise its large size would hinder further membrane proximity. In this scenario, Munc13–1 could not underlie changes in release probability directly, but its enhanced activity could lead to an increase in the number of preassembled SNARE complexes that might underlie the increases in release probability caused by up-regulation of DAG, by PDBu treatment or by the K630W mutation in a Ca^2+^ binding loop of the Munc13–2 C_2_B domain (Rhee et al., 2002; Shin et al., 2010). However, it is difficult to explain with this type of model why the H567K mutation in the C_1_ domain does not alter the vesicle refilling rate under resting conditions while increasing the Pvr (Rhee et al., 2002), and why PDBu changes the vesicle release probability without altering the RRP (Basu et al., 2007).

The distinct effects of Munc13 mutations on evoked release, RRP, vesicular release probability, synaptic responses to repetitive stimulation and PDBu-dependent facilitation [Figures 3–7 and (Rhee et al., 2002; Basu et al., 2007; Shin et al., 2010) are more readily explained by models postulating that Munc13–1 remains bound to the SNARE complex and/or other components of the release apparatus after SNARE complex assembly, forming part of the fusion complex. Such models predict at least two major actions for Munc13s, one in docking-priming (i.e. orchestrating SNARE complex assembly) and another in fusion. The length of the C_1_C_2_BMUN structure suggests that the Munc13–1 C-terminal region cannot remain in the central space between two membranes (as in Figure 9A) after the SNARE complex is even partially assembled. However, the activities of Munc13–1 in bridging the two membranes and mediating SNARE complex assembly, as well as potential downstream functions, could be performed with Munc13–1 located in the periphery of the membrane-membrane interface, with an arrangement where the large size of Munc13–1 does not hinder completion of SNARE complex assembly and membrane fusion (e.g. Figure 9B). With such an arrangement, reorientation of Munc13–1 upon binding to DAG and Ca^2+^-PIP_2_ could still underlie an increased activity in facilitating SNARE complex formation, but such reorientation could also lead to an optimization of the primed state that can lead more readily to membrane fusion upon Ca^2+^ influx than that observed without Munc13–1 activation. This separation of the roles of Munc13–1 into at least two steps can easily explain the differential effects of the V549E,L554E, R750E,K752E and N940W mutations in the different functional assays, including the finding that N940W impairs priming and evoked release, but enhances Munc13–1 function during 10 Hz trains or PDBu treatment (Figures 4–7).

Clearly, the primed fusion complex depicted in Figure 9B (top) might contain additional components such as Munc18–1, αSNAP, NSF, synaptotagmin-1 and complexins, among others. An attractive feature of this model is that it places the SNAREs on the periphery of the membrane-membrane interface rather than between the membranes, consistent with electron microscopy images of SNARE-mediated liposome fusion intermediates (Hernandez et al., 2012) and of presynaptic terminals showing practically direct contact between docked-primed vesicles and the plasma membrane [e.g. (Harlow et al., 2001)]. With this architecture, the SNARE complex could not induce fusion by bringing the vesicle and plasma membranes into proximity, as they are already in contact before Ca^2+^ influx, but could pull the two membranes radially outwards as assembly of the SNARE complex is completed (Figure 9B). In this case, fusion would result from the tension created in both membranes, which would favor transient formation of non-bilayer intermediates that might or might not resemble a stalk. Components of the fusion complex that are bound to the SNARE complex and bridge the membranes (e.g. Munc13–1 in Figure 9B) would play an important role to establish support points from which the SNAREs can exert their force on the membranes more efficiently.

As discussed in the results section, the strong effect of the R750E,K752E mutation in our fusion assays (Figure 8) suggests that our reconstitutions recapitulate better the mechanism of release during repetitive stimulation than release evoked by a single action potential. This proposal is supported by the findings that the tight Ca^2+^ dependence of membrane fusion in our reconstitution assays (Figure 8) arises largely from Ca^2+^ binding to the Munc13–1 C_2_B domain (Liu et al., 2016), and that disrupting the Ca^2+^-binding sites of the Munc13–2 C_2_B domain did not impair evoked release in rescue experiments but did impair release during repetitive stimulation (Shin et al., 2010). However, it is plausible that functional redundancy with other proteins and/or compensatory effects may have masked an actual role for the Munc13–2 C_2_B domain in Ca^2+^ sensing during evoked release, in cooperation with synaptotagmin-1. A primed state that contains Munc13 as key component bodes well for such a Ca^2+^-sensing role of the C_2_B domain, which is also suggested by the increased release probability caused by a K630W mutation in the Ca^2+^-binding loops of the Munc13–2 C_2_B domain (Shin et al., 2010) and may be reflected in the tight Ca^2+^-dependence of our fusion assays.

Clearly, further research is needed to test all these ideas, and it will be particularly critical to assess whether Munc13–1 is indeed part of the primed fusion complex and, if this is the case, how Munc13–1 binds to the SNAREs and other potential components of the complex. Regardless of these possibilities, the structure of C_1_C_2_BMUN presented here provides a fundamental framework to design and interpret future studies.

## Materials and methods

### Plasmids and recombinant proteins

To express rat Munc13–1 fragments encoding the C_1_C_2_BMUN and C_1_C_2_BMUNC_2_C regions (residues 529–1531 and 529–1735, respectively; both constructs have residues 1408–1452 from a flexible loop deleted), the corresponding DNA sequences were originated from full-length rat Munc13–1 (NM_022861, L756W, Δ1415–1437, Δ1533–1551, E1666G). C_1_C_2_BMUN was cloned into pFASTBacHTb (EcoRI, HindIII); C_1_C_2_BMUNC_2_C was cloned into pFASTBac(EcoRI, HindIII), which was modified by adding a GST tag and a TEV cleavage site in front of the EcoRI cloning site. The constructs were used to generate a baculovirus using the Bac-to-Bac system (Invitrogen; Waltham, MA). Insect cells (sf9) were infected with the baculovirus, harvested about 72–96 hr post-infection, and re-suspended in lysis buffers C_1_C_2_BMUN (50 mM Tris pH8.0, 250 mM NaCl, 10 mM imidazole); C_1_C_2_BMUNC_2_C (50 mM Tris pH8.0, 250 mM NaCl, 1 mM TCEP). Cells were lysed and centrifuged at 18,000 rpm for 45 min. The clear supernatant of C_1_C_2_BMUN was incubated with Ni-NTA resin at 4°C for 2 hr, then the beads were washed with: (i) lysis buffer; (ii) lysis buffer containing 1% Triton X-100; (iii) lysis buffer containing 1M NaCl; and (iv) lysis buffer. The protein was eluted in lysis buffer with 150 mM imidazole, incubated with TEV to remove the His-tag, and purified by ion exchange chromatography. The clear supernatant of C_1_C_2_BMUNC_2_C was incubated with GST agarose at room temperature for 2 hr. The beads were washed with: (i) lysis buffer; (ii) lysis buffer containing 1% Triton X-100; (iii) lysis buffer containing 1M NaCl; and (iv) lysis buffer. The protein was treated with TEV protease on the GST agarose at 22°C for 2 hr. Both C_1_C_2_BMUN and C_1_C_2_BMUNC_2_C were further purified via gel filtration chromatography and were concentrated to 3–4 mg/ml for storage in 10 mM Tris buffer (pH 8.0) containing 10% glycerol, 5 mM TCEP and 250 mM NaCl. The C_1_C_2_BMUNC_2_C mutants were generated by site-directed mutagenesis, and purified as the WT fragment. The finding that the mutants eluted at the same volumes as WT C_1_C_2_BMUNC_2_C in gel filtration and did not exhibit a higher tendency to degradation strongly suggest that the mutations do not cause folding problems.

### Crystallization and X-ray diffraction data collection

Rat Munc13–1 C_1_C_2_BMUN (529-1407, 1453-1531) in 0.01 M Tris (pH 8.0), 0.25 M NaCl, 10% (v/v) glycerol and 5 mM TCEP was concentrated to 4–6 mg/ml for crystallization using the sitting drop vapor diffusion method. Drops in a ratio of 1 μl protein to 1 μl well solution were equilibrated against 200 μl 0.1 M Tris (pH 8.0–8.5), 0.2 M LiCl, 12% (v/v) PEG 10,000 at 20°C. Multiple crystals appeared spontaneously in 3 days and were used for dilution microseeding. Drops in a ratio of 1 μl protein to 1–2 μl well solution premixed with crystal seeds were equilibrated against 200 μl 0.1 M Tris (pH 8.0–8.5), 0.2 M LiCl, 10–12% (v/v) PEG 10,000 at 20°C, and crystals were harvested within 10 days. Tantalum derivatized crystals were obtained by overnight incubation with solid Na_2_Ta_6_Br_12_ in mother liquor drops that contained pre-grown single C_1_C_2_BMUN crystals. Crystals were cryoprotected by successive transfer in increasing steps of 5% ethylene glycol to a final solution of 20–25% (v/v) ethylene glycol, 0.1 M Tris (pH 8.0), 0.2 M LiCl, 0.15 M NaCl, 10%(v/v) glycerol, 5 mM TCEP and flash-cooled in liquid nitrogen.

Native C_1_C_2_BMUN crystals exhibited the symmetry of space group C2 with unit-cell parameters of a = 176.1 Å, b = 86.4 Å, c = 202.1 Å and β = 115.5° and contained two molecules of C_1_C_2_BMUN per asymmetric unit. C_1_C_2_BMUN crystals displayed strong anisotropy and were highly non-isomorphous. Native C_1_C_2_BMUN crystals diffracted to a d_min_ of 3.35 Å when exposed to synchrotron radiation. All diffraction data were collected at beamline 19-ID (SBC-CAT) at the Advanced Photon Source (Argonne National Laboratory, Argonne, IL, USA) and processed with *HKL3000* (Minor et al., 2006), with applied corrections for effects resulting from absorption in a crystal and for radiation damage (Borek et al., 2003; Otwinowski et al., 2003), the calculation of an optimal error model, and corrections to compensate the phasing signal for a radiation-induced increase of non-isomorphism within the crystal (Borek et al., 2010, 2013). These corrections were crucial for successful phasing. Initial phases for C_1_C_2_BMUN were obtained by molecular replacement (MR) with *Phaser* (McCoy et al., 2007) using the crystal structure of the N-terminally truncated MUN BCD domain (PDB code: 4Y21) (Yang et al., 2015) with the coordinates for several loops and residues at the N- and C-termini removed (residues 942–947, 1038–1041, 1193–1196, 1342–1353, 1404–1409, 1452–1469, 1515–1523) as the search model. One C2B domain was located by MR using the crystal structure of the calcium-free C2B domain (PDB code: 3 KWT) as a search model (Shin et al., 2010). Phasing using MR versus the native dataset stalled at this point, and an iterative MR-SAD/rigid body refinement procedure was then adopted. MR-SAD phases calculated in *Phaser* from a tantalum bromide dataset collected at the tantalum LIII edge revealed interpretable density for missing helices H7-H9, which were modeled initially with a polyalanine sequence and rigid body refined in *Phenix* (Adams et al., 2010). The updated model was placed in the native cell via MR in *Phaser* and rigid-body refined in *Phenix.* Density modification using two-fold non-crystallographic symmetry was performed in *Parrot* (Cowtan, 2010), and automated model rebuilding of only the newly added four helices of each MUN domain was performed in *Buccaneer* (Cowtan, 2006). Multiple cycles of alternating this procedure between the tantalum and native datasets allowed the modeling and sequence assignment to helices H1-H9, and the placement of the second C2B domain by MR. Subsequently, both C1 domains were located by MR using the NMR structure (PDB code: 1Y8F) as a search model (Shen et al., 2005), and the locations were confirmed by an anomalous difference map calculated from a dataset collected at the zinc K-edge (Figure 1—figure supplement 1). Iterative model building and refinement were performed with *COOT* and *Phenix*, respectively (Emsley and Cowtan, 2004).

Restraints used in the initial cycles of model refinement included non-crystallographic symmetry, secondary structure and reference models for the C1, C2B and MUN domains. As the coordinates for the MUN domain deposited in the PDB (ID 4Y21) had incorrect sequence numbering and exhibited a high level of side chain outliers, a high clashscore and high overall score in *MolProbity* (Chen et al., 2010), the coordinates for the MUN domain were re-refined versus the deposited structure factors and the sequence numbering was corrected prior to use as a reference model for restrained refinement of the C1C2BMUN model. The reference model restraints were removed for the final cycles of refinement. A superposition of chains A and B yield a root mean square deviation (r.m.s.d.) of 1.51 Å for 809 aligned Cα carbons (Figure 1—figure supplement 2). The final model for C1C2BMUN (R_work_ = 25.4%, R_free_ = 29.0%) contained 1685 residues in two monomers, 4 Zn^2+^ and 2 Cl^-^ ions. The higher-than-average R_free_ value is probably due to the relative dearth of lattice contacts (Figure 1—figure supplement 3) for the CD subdomains of MUN chain A of C1C2BMUN, as evidenced by weak electron density and high average thermal displacement factors (ADP) (151.2 Å^2^) for those subdomains (residues 1255–1517) (Figure 1—figure supplement 4). Due to the high ADP values for the CD subdomain of chain A, the authors recommend that interpretation of the CD subdomain should be performed on residues in chain B. The density for the remaining domains of chain A as well as chain B of C1C2BMUN is strong and well connected; in fact, the average ADPs for residues 541–950 are lower for chain A (41.0 Å^2^) than chain B (62.4 Å^2^). Omit maps for regions where site directed mutations were made are shown in Figure 1—figure supplement 5. A Ramachandran plot generated with *MolProbity* (Chen et al., 2010) indicated that 92.6% of all protein residues are in the most favored regions and 1.2% in disallowed regions. The majority of the outliers in the Ramachandran plot are located in surface loops with weak electron density that connect domains or secondary structural elements. Data collection and structure refinement statistics are summarized in Table 1. The coordinates of the C_1_C_2_BMUN structure and of the refined MUN domain structure have been deposited in the Protein Data Bank with accession numbers 5UE8 and 5UF7, respectively.

### Lentiviral constructs

The cDNAs of Munc13–1 full length and Munc13–1 V549E,L554E, Munc13–1 R750E,K752E and Munc13–1 N940W were generated from rat Munc13–1 (Basu et al., 2005) by PCR amplification. The reverse primer harbors a 3xFLAG sequence (Sigma-Aldrich, Hamburg, Germany) to allow expression analysis. The corresponding PCR products were fused to a P2A linker (Kim et al., 2011) after a nuclear localized GFP sequence into the lentiviral shuttle vector, which allows a bicistronic expression of NLS-GFP and the Munc13–1-Flag protein under the control of a human *synapsin-1* promotor. Concentrated lentiviral particles were prepared as described (Lois et al., 2002).

### Autaptic hippocampal neuronal cultures and lentiviral infection

Animal welfare committees of Charité Medical University and the Berlin state government Agency for Health and Social Services approved all protocols for animal maintenance and experiments (license no. T 0220/09). Hippocampi were dissected from embryonic day 18.5 Munc13 1/2 DKO mouse and enzymatically treated with 25 units/ml of papain for 45 min at 37°C. After enzyme digestion, hippocampi were mechanically dissociated and the neuron suspension was plated onto astrocytes microislands at a final density of 300 cells cm^−2^. Neurons were infected 24 hr after plating with the lentiviral rescue constructs and incubated at 37°C and 5% CO_2_ for 13–16 days.

### Electrophysiology

Whole-cell voltage clamp recordings were done at room temperature in 13–16 days in vitro (DIV) autaptic hippocampal Munc13- 1/2 DKO neurons expressing Munc13–1 WT, Munc13–1 V549E,L554E, Munc13–1 R750E,K752E or Munc13–1 N940W. Synaptic currents were monitored using a Multiclamp 700B amplifier (Molecular Devices). The series resistance was compensated by 70% and only cells with series resistances <10 MΩ were analyzed. Data were acquired using Clampex 10 software (Molecular Devices, Sunnyvale, CA) at 10 kHz and filtered using a low-pass Bessel filter at 3 kHz. Borosilicate glass pipettes with a resistance between 2 and 3.5 MΩ were used. Pipettes were filled with internal recording solution contained the following (in mM): 136 KCl, 17.8 HEPES, 1 EGTA, 4.6 MgCl_2_, 4 Na_2_ATP, 0.3 Na_2_GTP, 12 creatine phosphate, and 50 U/ml phosphocreatine kinase; 300 mOsm; pH 7.4. During recordings, neurons were continuously perfused with standard extracellular solution including the following (in mM): 140 NaCl, 2.4 KCl, 10 HEPES, 10 glucose, 2 CaCl_2_, 4 MgCl_2_; 300 mOsm; pH 7.4. Spontaneous release was measured by recording miniature EPSCs for 30 s at −70 mV. To detect false-positive events 3 mM of kynurenic acid was applied for an equal amount of time. Action potential-evoked EPSCs were triggered by 2 ms somatic depolarization from −70 to 0 mV. The size of the readily-releasable pool (RRP) was determined by the application with a fast flow system of 500 mM sucrose added to the standard extracellular solution for 5 s. Evoked sucrose responses are characterized by a transient inward current followed by a steady state current. The steady state component represents refilling of primed vesicles and was used to define the baseline. The area under the baseline in the transient curve component was quantified to determine the total charge released by the RRP (Rosenmund and Stevens, 1996). The vesicular release probability (*p_vr_*) was calculated by dividing the EPSC charge by the RRP charge.

The paired-pulse stimulation protocol contained two inductions of an AP at an interval of 25 ms (40 Hz). The paired-pulse ratio was calculated by dividing the amplitude of the second EPSC by the amplitude of the first. To analyze release induced by a high frequency stimulation train, EPSCs were evoked at a frequency of 10 Hz for 5 s. To define the quantitative relationship between Pvr and the steady state EPSC amplitudes during a 10 Hz action potential train, we recorded EPSCs and sucrose evoked responses from wildtype neurons. EPSCs and EPSC trains were recorded in external solutions containing 0.5, 1, 2 and 4 mM calcium and 4 mM magnesium. The hypertonic sucrose solution for all responses contained 2 mM calcium and 4 mM magnesium. The Pvr was computed and correlated with the mean amplitude of the last 10 EPSCs of the 10 Hz AP train. Phorbol ester experiments were monitored in the same cell, EPSC amplitudes were recorded at 0.2 Hz for 30 s in the absence of 1 µM PDBu and the following 30 s in its presence. To dissect how an AP train modulates synaptic output, we examined RRP and release probability separately by probing EPSC amplitude and RRP size 2 s following the 10 Hz train. We then compared the relative changes in RRP and EPSC to the corresponding EPSC and RRP values preceding the AP train. An approximately equal number of cells were recorded from control and experimental groups per day from 3 to 4 consecutive days. The present dataset was acquired from three separate cultures. To minimize variability between cultures the values of mEPSC, EPSC and RRP from each experimental groups of recording were normalized to the mean value of the WT group for each culture. Data were analyzed offline using Axograph X (Axograph Scientific, Sidney, Australia) and the values were normalized to the WT group. Data summation and statistical analyses were performed using Prism 7 (GraphPad). Significance and p values were determined by comparison of each mutant group with the Munc13–1 WT using the unpaired Student's *t* test: Mann-Whitney.

### Munc13–1 protein expression level quantification by Western blot

Hippocampal neurons from Munc13-1/2 DKO expressing Munc13–1 full length, Munc13–1 V549E,L554E, Munc13–1 R750E,K752E and Munc13–1 N940W mutants were lysed after 15 DIV at 4°C with RIPA lysis buffer including protease inhibitor cocktail-complete mini (Roche Diagnostics, Berlin, Germany). Equal amounts of proteins from the lysates of the four different groups were mixed with Laemmli sample buffer containing 0.1 M DTT, and boiled 5 min at 99°C. Protein lysates were separated on SDS polyacrylamide gels (4–8%% SDS-PAGE) and transferred to a polyvinyl difluoride (PVDF) membrane. Membranes were blocked for 1 hr with 5% skim milk in TBST and incubated at 4°C over night with primary antibodies: anti-Flag M2 (F1804; Sigma-Aldrich), and anti-Living Colors GFP (632375; Clontech, Mountain View, CA). Secondary antibodies were horseradish peroxidase-conjugated (Jackson ImmunoResearch, West Grove, PA). The immunoreactive proteins were detected by ECL Plus Western Blotting Detection Reagents (GE Healthcare Biosciences, Pittsuburgh, PA) in a Fusion FX7 detection system (Vilber Lourmat, Eberhardzell, Germany). Data were collected from three separate Munc13-1/2 DKO cultures and analyzed offline using ImageJ.

## Additional files

### Major datasets

The following datasets were generated:

Tomchick DR,Rizo J,Xu J,2016,C1C2BMUN structure,http://www.rcsb.org/pdb/explore/explore.do?structureId=5UE8,Publicly available at the Protein Data Bank (accession no: 5UE8)

Tomchick DR,Rizo J,Xu J,2016,Refined MUN domain structure,http://www.rcsb.org/pdb/explore/explore.do?structureId=5UF7,Publicly available at the Protein Data Bank (accession no: 5UF7)

## References

[bib1] Adams PD, Afonine PV, Bunkóczi G, Chen VB, Davis IW, Echols N, Headd JJ, Hung LW, Kapral GJ, Grosse-Kunstleve RW, McCoy AJ, Moriarty NW, Oeffner R, Read RJ, Richardson DC, Richardson JS, Terwilliger TC, Zwart PH (2010). PHENIX: a comprehensive Python-based system for macromolecular structure solution. Acta Crystallographica Section D Biological Crystallography.

[bib2] Aravamudan B, Fergestad T, Davis WS, Rodesch CK, Broadie K (1999). Drosophila UNC-13 is essential for synaptic transmission. Nature Neuroscience.

[bib3] Augustin I, Rosenmund C, Südhof TC, Brose N (1999). Munc13-1 is essential for fusion competence of glutamatergic synaptic vesicles. Nature.

[bib4] Banerjee A, Barry VA, DasGupta BR, Martin TF (1996). N-Ethylmaleimide-sensitive factor acts at a prefusion ATP-dependent step in Ca2+-activated exocytosis. Journal of Biological Chemistry.

[bib5] Basu J, Betz A, Brose N, Rosenmund C (2007). Munc13-1 C1 domain activation lowers the energy barrier for synaptic vesicle fusion. Journal of Neuroscience.

[bib6] Basu J, Shen N, Dulubova I, Lu J, Guan R, Guryev O, Grishin NV, Rosenmund C, Rizo J (2005). A minimal domain responsible for Munc13 activity. Nature Structural & Molecular Biology.

[bib7] Betz A, Thakur P, Junge HJ, Ashery U, Rhee JS, Scheuss V, Rosenmund C, Rettig J, Brose N (2001). Functional interaction of the active zone proteins Munc13-1 and RIM1 in synaptic vesicle priming. Neuron.

[bib8] Borek D, Cymborowski M, Machius M, Minor W, Otwinowski Z (2010). Diffraction data analysis in the presence of radiation damage. Acta Crystallographica Section D Biological Crystallography.

[bib9] Borek D, Dauter Z, Otwinowski Z (2013). Identification of patterns in diffraction intensities affected by radiation exposure. Journal of Synchrotron Radiation.

[bib10] Borek D, Minor W, Otwinowski Z (2003). Measurement errors and their consequences in protein crystallography. Acta Crystallographica Section D Biological Crystallography.

[bib11] Brose N, Hofmann K, Hata Y, Südhof TC (1995). Mammalian homologues of Caenorhabditis elegans unc-13 gene define novel family of C2-domain proteins. Journal of Biological Chemistry.

[bib12] Chan YH, van Lengerich B, Boxer SG (2009). Effects of linker sequences on vesicle fusion mediated by lipid-anchored DNA oligonucleotides. PNAS.

[bib13] Chen VB, Arendall WB, Headd JJ, Keedy DA, Immormino RM, Kapral GJ, Murray LW, Richardson JS, Richardson DC (2010). *MolProbity*: all-atom structure validation for macromolecular crystallography. Acta Crystallographica Section D Biological Crystallography.

[bib14] Cowtan K (2006). The *buccaneer* software for automated model building. 1. Tracing protein chains. Acta Crystallographica. Section D, Biological Crystallography.

[bib15] Cowtan K (2010). Recent developments in classical density modification. Acta Crystallographica Section D Biological Crystallography.

[bib16] Deng L, Kaeser PS, Xu W, Südhof TC (2011). RIM proteins activate vesicle priming by reversing autoinhibitory homodimerization of Munc13. Neuron.

[bib17] Dulubova I, Lou X, Lu J, Huryeva I, Alam A, Schneggenburger R, Südhof TC, Rizo J (2005). A Munc13/RIM/Rab3 tripartite complex: from priming to plasticity?. The EMBO Journal.

[bib18] Dulubova I, Sugita S, Hill S, Hosaka M, Fernandez I, Südhof TC, Rizo J (1999). A conformational switch in syntaxin during exocytosis: role of munc18. The EMBO Journal.

[bib19] Emsley P, Cowtan K (2004). Coot: model-building tools for molecular graphics. Acta Crystallographica. Section D, Biological Crystallography.

[bib20] Emsley P, Lohkamp B, Scott WG, Cowtan K (2010). Features and development of coot. Acta Crystallographica. Section D, Biological Crystallography.

[bib21] Ernst JA, Brunger AT (2003). High resolution structure, stability, and synaptotagmin binding of a truncated neuronal SNARE complex. Journal of Biological Chemistry.

[bib22] Hammarlund M, Palfreyman MT, Watanabe S, Olsen S, Jorgensen EM (2007). Open syntaxin docks synaptic vesicles. PLoS Biology.

[bib23] Hanson PI, Roth R, Morisaki H, Jahn R, Heuser JE (1997). Structure and conformational changes in NSF and its membrane receptor complexes visualized by quick-freeze/deep-etch Electron microscopy. Cell.

[bib24] Harlow ML, Ress D, Stoschek A, Marshall RM, McMahan UJ (2001). The architecture of active zone material at the frog's neuromuscular junction. Nature.

[bib25] Hernandez JM, Stein A, Behrmann E, Riedel D, Cypionka A, Farsi Z, Walla PJ, Raunser S, Jahn R (2012). Membrane fusion intermediates via directional and full assembly of the SNARE complex. Science.

[bib26] Imig C, Min SW, Krinner S, Arancillo M, Rosenmund C, Südhof TC, Rhee J, Brose N, Cooper BH (2014). The morphological and molecular nature of synaptic vesicle priming at presynaptic active zones. Neuron.

[bib27] Jahn R, Fasshauer D (2012). Molecular machines governing exocytosis of synaptic vesicles. Nature.

[bib28] Junge HJ, Rhee JS, Jahn O, Varoqueaux F, Spiess J, Waxham MN, Rosenmund C, Brose N (2004). Calmodulin and Munc13 form a Ca2+ sensor/effector complex that controls short-term synaptic plasticity. Cell.

[bib29] Kim JH, Lee SR, Li LH, Park HJ, Park JH, Lee KY, Kim MK, Shin BA, Choi SY (2011). High cleavage efficiency of a 2A peptide derived from porcine teschovirus-1 in human cell lines, zebrafish and mice. PLoS One.

[bib30] Li W, Ma C, Guan R, Xu Y, Tomchick DR, Rizo J (2011). The crystal structure of a Munc13 C-terminal module exhibits a remarkable similarity to vesicle tethering factors. Structure.

[bib31] Liu X, Seven AB, Camacho M, Esser V, Xu J, Trimbuch T, Quade B, Su L, Ma C, Rosenmund C, Rizo J (2016). Functional synergy between the Munc13 C-terminal C1 and C2 domains. eLife.

[bib32] Lois C, Hong EJ, Pease S, Brown EJ, Baltimore D (2002). Germline transmission and tissue-specific expression of transgenes delivered by lentiviral vectors. Science.

[bib33] Lu J, Machius M, Dulubova I, Dai H, Südhof TC, Tomchick DR, Rizo J (2006). Structural basis for a Munc13-1 homodimer to Munc13-1/RIM heterodimer switch. PLoS Biology.

[bib34] Ma C, Li W, Xu Y, Rizo J (2011). Munc13 mediates the transition from the closed syntaxin-Munc18 complex to the SNARE complex. Nature Structural & Molecular Biology.

[bib35] Ma C, Su L, Seven AB, Xu Y, Rizo J (2013). Reconstitution of the vital functions of Munc18 and Munc13 in neurotransmitter release. Science.

[bib36] Mayer A, Wickner W, Haas A (1996). Sec18p (NSF)-driven release of Sec17p (alpha-SNAP) can precede docking and fusion of yeast vacuoles. Cell.

[bib37] McCoy A, Grosse-Kunstleve RW, Adams PD, Winn MD, Storoni LC, Read RJ (2007). *Phaser* crystallographic software. Journal of Applied Crystallography.

[bib38] Minor W, Cymborowski M, Otwinowski Z, Chruszcz M (2006). HKL-3000: the integration of data reduction and structure solution--from diffraction images to an initial model in minutes. Acta Crystallographica Section D Biological Crystallography.

[bib39] Misura KM, Scheller RH, Weis WI (2000). Three-dimensional structure of the neuronal-Sec1-syntaxin 1a complex. Nature.

[bib40] Otwinowski Z, Borek D, Majewski W, Minor W (2003). Multiparametric scaling of diffraction intensities. Acta Crystallographica Section a Foundations of Crystallography.

[bib41] Pei J, Ma C, Rizo J, Grishin NV (2009). Remote homology between Munc13 MUN domain and vesicle tethering complexes. Journal of Molecular Biology.

[bib42] Poirier MA, Xiao W, Macosko JC, Chan C, Shin YK, Bennett MK (1998). The synaptic SNARE complex is a parallel four-stranded helical bundle. Nature Structural Biology.

[bib43] Regehr WG (2012). Short-term presynaptic plasticity. Cold Spring Harbor Perspectives in Biology.

[bib44] Rhee JS, Betz A, Pyott S, Reim K, Varoqueaux F, Augustin I, Hesse D, Südhof TC, Takahashi M, Rosenmund C, Brose N (2002). Beta phorbol ester- and diacylglycerol-induced augmentation of transmitter release is mediated by Munc13s and not by PKCs. Cell.

[bib45] Richmond JE, Davis WS, Jorgensen EM (1999). UNC-13 is required for synaptic vesicle fusion in C. elegans. Nature Neuroscience.

[bib46] Richmond JE, Weimer RM, Jorgensen EM (2001). An open form of syntaxin bypasses the requirement for UNC-13 in vesicle priming. Nature.

[bib47] Rizo J, Rosenmund C (2008). Synaptic vesicle fusion. Nature Structural & Molecular Biology.

[bib48] Rizo J, Xu J (2015). The synaptic vesicle release machinery. Annual Review of Biophysics.

[bib49] Rodríguez-Castañeda F, Maestre-Martínez M, Coudevylle N, Dimova K, Junge H, Lipstein N, Lee D, Becker S, Brose N, Jahn O, Carlomagno T, Griesinger C (2010). Modular architecture of Munc13/calmodulin complexes: dual regulation by Ca2+ and possible function in short-term synaptic plasticity. The EMBO Journal.

[bib50] Rosenmund C, Sigler A, Augustin I, Reim K, Brose N, Rhee JS (2002). Differential control of vesicle priming and short-term plasticity by Munc13 isoforms. Neuron.

[bib51] Rosenmund C, Stevens CF (1996). Definition of the readily releasable pool of vesicles at hippocampal synapses. Neuron.

[bib52] Shen N, Guryev O, Rizo J (2005). Intramolecular occlusion of the diacylglycerol-binding site in the C1 domain of munc13-1. Biochemistry.

[bib53] Shin OH, Lu J, Rhee JS, Tomchick DR, Pang ZP, Wojcik SM, Camacho-Perez M, Brose N, Machius M, Rizo J, Rosenmund C, Südhof TC (2010). Munc13 C2B domain is an activity-dependent Ca2+ regulator of synaptic exocytosis. Nature Structural & Molecular Biology.

[bib54] Sutton RB, Fasshauer D, Jahn R, Brunger AT (1998). Crystal structure of a SNARE complex involved in synaptic exocytosis at 2.4 A resolution. Nature.

[bib55] Söllner T, Bennett MK, Whiteheart SW, Scheller RH, Rothman JE (1993). A protein assembly-disassembly pathway in vitro that may correspond to sequential steps of synaptic vesicle docking, activation, and fusion. Cell.

[bib56] Südhof TC, Rothman JE (2009). Membrane fusion: grappling with SNARE and SM proteins. Science.

[bib57] Südhof TC (2013). Neurotransmitter release: the last millisecond in the life of a synaptic vesicle. Neuron.

[bib58] Varoqueaux F, Sigler A, Rhee JS, Brose N, Enk C, Reim K, Rosenmund C (2002). Total arrest of spontaneous and evoked synaptic transmission but normal synaptogenesis in the absence of Munc13-mediated vesicle priming. PNAS.

[bib59] Weimer RM, Gracheva EO, Meyrignac O, Miller KG, Richmond JE, Bessereau JL (2006). UNC-13 and UNC-10/rim localize synaptic vesicles to specific membrane domains. Journal of Neuroscience.

[bib60] Yang X, Wang S, Sheng Y, Zhang M, Zou W, Wu L, Kang L, Rizo J, Zhang R, Xu T, Ma C (2015). Syntaxin opening by the MUN domain underlies the function of Munc13 in synaptic-vesicle priming. Nature Structural & Molecular Biology.

[bib61] Yu IM, Hughson FM (2010). Tethering factors as organizers of intracellular vesicular traffic. Annual Review of Cell and Developmental Biology.

[bib62] Zhang G, Kazanietz MG, Blumberg PM, Hurley JH (1995). Crystal structure of the cys2 activator-binding domain of protein kinase C Delta in complex with phorbol ester. Cell.

[bib63] Zick M, Wickner WT (2014). A distinct tethering step is vital for vacuole membrane fusion. eLife.

[bib64] Zucchi PC, Zick M (2011). Membrane fusion catalyzed by a rab, SNAREs, and SNARE chaperones is accompanied by enhanced permeability to small molecules and by lysis. Molecular Biology of the Cell.

